# Water-Restrained Hydrogel Electrolytes with Repulsion-Driven Cationic Express Pathways for Durable Zinc-Ion Batteries

**DOI:** 10.1007/s40820-025-01704-5

**Published:** 2025-03-19

**Authors:** Dewu Lin, Yushuang Lin, Ruihong Pan, Jiapei Li, Anquan Zhu, Tian Zhang, Kai Liu, Dongyu Feng, Kunlun Liu, Yin Zhou, Chengkai Yang, Guo Hong, Wenjun Zhang

**Affiliations:** 1 Department of Materials Science and Engineering & Center of Super-Diamond and Advanced Films (COSDAF), City University of Hong Kong, Kowloon, 999077 People’s Republic of China; 2 College of Materials Science and Engineering, Fuzhou University, Fuzhou, 350108 People’s Republic of China; 3 The Shenzhen Research Institute, City University of Hong Kong, Shenzhen, 518057 People’s Republic of China

**Keywords:** Zinc-ion battery, Hydrogel electrolyte, Cation conduction, Ionic repulsion, Water state

## Abstract

The development of flexible zinc-ion batteries (ZIBs) faces a three-way trade-off among the ionic conductivity, Zn^2+^ mobility, and the electrochemical stability of hydrogel electrolytes. To address this challenge, we designed a cationic hydrogel named PAPTMA to holistically improve the reversibility of ZIBs. The long cationic branch chains in the polymeric matrix construct express pathways for rapid Zn^2+^ transport through an ionic repulsion mechanism, achieving simultaneously high Zn^2+^ transference number (0.79) and high ionic conductivity (28.7 mS cm^−1^). Additionally, the reactivity of water in the PAPTMA hydrogels is significantly inhibited, thus possessing a strong resistance to parasitic reactions. Mechanical characterization further reveals the superior tensile and adhesion strength of PAPTMA. Leveraging these properties, symmetric batteries employing PAPTMA hydrogel deliver exceeding 6000 h of reversible cycling at 1 mA cm^−2^ and maintain stable operation for 1000 h with a discharge of depth of 71%. When applied in 4 × 4 cm^2^ pouch cells with MnO_2_ as the cathode material, the device demonstrates remarkable operational stability and mechanical robustness through 150 cycles. This work presents an eclectic strategy for designing advanced hydrogels that combine high ionic conductivity, enhanced Zn^2+^ mobility, and strong resistance to parasitic reactions, paving the way for long-lasting flexible ZIBs.

**Supplementary Information:**

The online version contains supplementary material available at 10.1007/s40820-025-01704-5.

## Highlights

- A novel cationic hydrogel electrolyte is prepared to address a significant challenge of balancing the tripartite trade-offs of hydrogel properties.
- Cationic express pathways enable fast and selective Zn^2+^ transport through dynamic ionic repulsion, achieving high ionic conductivity (28.7 mS cm^−1^) and Zn^2+^ transference number (0.79).
- The hydrogel demonstrates exceptional cycling stability across − 15 to 60 °C, showcasing great potential for practical flexible battery applications.

**Supplementary Information:**

The online version contains supplementary material available at 10.1007/s40820-025-01704-5.

## Graphical abstract

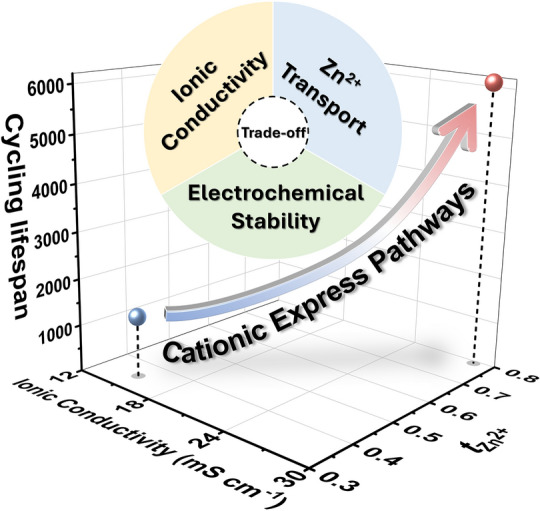

## Introduction

Rechargeable zinc-ion batteries (ZIBs) have been considered a promising solution for stationary energy storage and power supply of flexible devices due to their high theoretical capacity (820 mAh mg^−1^ and 5855 mAh cm^−3^), low cost (approximately $2 ~ 4 per kg for zinc), and the safety of aqueous electrolytes [1–3] However, despite these advantages, the development of ZIBs is impeded by several critical issues. In particular, long-lasting parasitic reactions occur between the Zn anode and the aqueous environment, including acidic corrosion, hydrogen evolution reaction (HER), and dendritic growth, which are ascribed to the chemical instability of Zn in water [4–6]. These parasitic reactions, induced by highly reactive water, would severely degrade the operating lifespan of ZIBs. In comparison with liquid electrolytes, hydrogel electrolytes, with their reduced free-state water content, are expected to effectively suppress these parasitic reactions. Additionally, the unique properties of hydrogels, such as deformability and self-healing, expand their applications in flexible energy storage devices [7–11]. Therefore, quasi-solid-state ZIBs employing hydrogel electrolytes become a promising alternative.

Various synthetic and natural polymeric matrices, *e.g.*, polyacrylamide (PAM) [12], polyvinyl alcohol [13], xanthan gum [14], and carrageenan [15], have been developed to enhance the electrochemical performance of ZIBs [16]. Hydrogels could be modified to further improve their performance, mainly targeting three objectives. (1) Improving ionic conductivity: Enhancing ionic conductivity is crucial for fast interfacial reaction kinetics [17–21]. For example, Yang et al. developed a supramolecular zwitterionic hydrogel electrolyte with a record-high ionic conductivity (*σ*) of 48 mS cm^−1^ through molecular engineering [20]. (2) Increasing Zn^2+^ mobility: Achieving high Zn^2+^ mobility is essential to prevent the formation of dendrite and loose zinc hydroxide [22–24]. Sun et al. built cationic channels in hydrogels by incorporating ring-shaped α-cyclodextrins with liner polymers, achieving an exceptional Zn^2+^ transference number $\left( {t_{{{\text{Zn}}^{2 + } }} } \right)$ of 0.92 [24]. 3) Reducing water content: Minimizing water content in hydrogel further inhibits parasitic reactions [25]. A lean-water hydrogel with only 20% water content has been fabricated by removing excess free-state water, demonstrating an expanded electrochemical stability window and high resistance to parasitic reactions [26].

Despite these advancements toward a single objective, complex trade-offs among these three targets persist. For instance, achieving a high $t_{{{\text{Zn}}^{2 + } }}$ (> 0.9) through covalent bonding of anions to polymer chains usually results in reduced overall *σ* (< 5 mS cm^−1^) [27, 28]. Conversely, water-rich hydrogels with high *σ* (> 25 mS cm^−1^) exhibit mediocre $t_{{{\text{Zn}}^{2 + } }}$ (< 0.6) and limited electrochemical stability [14]. Moreover, lean-water hydrogels, while offering excellent stability, typically show low *σ* (< 5 mS cm^−1^) [26]. High *σ* is often accompanied by high water reactivity, which could trigger hydrogen evolution reactions and dendritic growth. Till now, devising a comprehensive strategy that balances these targets remains a significant challenge.

**Fig. 1:**
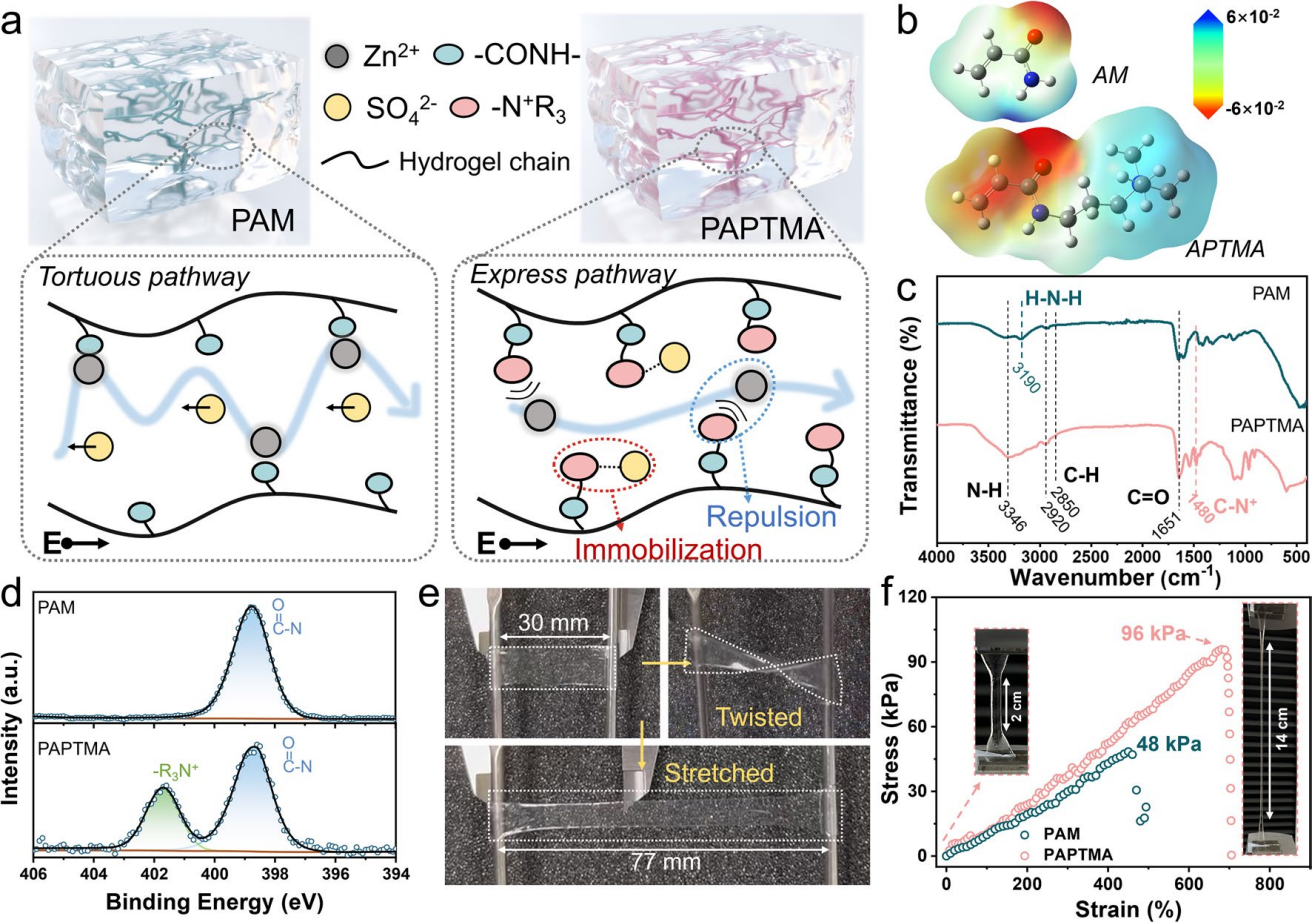
Morphology and properties of PAPTMA hydrogel electrolytes. **a** Schematics of the ion transport within PAM and PAPTMA hydrogels. **b** Electrostatic potential distributions simulated for AM and APTMA monomers, unit: Hartree. **c** FTIR spectra and **d** N1*s* XPS profiles of PAM and PAPTMA hydrogels. **e** Optical photographs showing the twisting and stretching behaviors of PAPTMA hydrogel. **f** Tensile tests of PAM and PAPTMA hydrogels

In this work, we have developed a unique ‘trifunctional’ strategy that simultaneously addresses three critical challenges in flexible AZIBs through the design of a cationic hydrogel electrolyte (designated as PAPTMA) using 3-acrylamidopropyl trimethylammonium chloride. The quaternary ammonium (–N^+^R_3_) branch chains construct pathways that facilitate the fast transfer of Zn^2+^ ions through ionic repulsion interaction, unlike the tortuous hopping mechanisms in conventional PAM hydrogels (Fig. 1a). Particularly, due to the apparent increase in the interfacial area between polymer and surrounding electrolyte, along with the presence of hydrophilic –N^+^R_3_ groups, free-state water molecules in the hydrogel were primarily converted to interfacial bound water, which effectively mitigated water-induced parasitic reactions without compromising ionic conductivity and Zn^2+^ transport. A systematic evaluation of by-products from parasitic reactions, specifically Zn_4_(OH)_6_SO_4_·5H_2_O (ZSH) and H_2_, confirmed the excellent electrochemical stability of our cationic hydrogel. Consequently, the poly-APTMA (PAPTMA) hydrogel electrolyte achieved an outstanding balance among $t_{{{\text{Zn}}^{2 + } }}$ (0.79), *σ* (28.7 mS cm^−1^), and electrochemical stability. As a result, Zn symmetric cells employing PAPTMA hydrogel delivered stable cycling lifespans exceeding 6000 h at 1 mA cm^−2^ and 1200 h at 4 mA cm^−2^. Furthermore, a 4 × 4 cm^2^ pouch cell also demonstrated a durable capacity of 150 mAh g^−1^ under bending deformation ranging from 0° to 360°. This water reactivity-restrained PAPTMA hydrogel electrolyte, with its cation express pathways, provides a new opportunity for the rational design of advanced hydrogel electrolytes with balanced properties.

## Experimental Section

### Preparation of Hydrogel Electrolyte

Typically, 1.0 g acrylamide (AM) and 2.30 g ZnSO_4_·7H_2_O were first dissolved into 4 mL deionized (DI) water by stirring. Subsequently, 20 mg N, N′-methylenebisacrylamide (MBA) was added, and the mixture was stirred until a transparent solution was obtained. After that, 100 μL potassium persulfate aqueous solution (2 M) was added, followed by transferring the precursor solution into a glass mold (100 × 100 × 0.5 mm^3^). The gelation process was carried out at 60 °C for 2 h. For the preparation of other hydrogel electrolytes in this work, the detailed dosages of all reagents are listed in Table S1 without changing the operation process.

### Material Characterization

Scanning electron microscopy (SEM) was performed using QUATTRO S (Thermo Fisher Scientific). X-ray photoelectron spectroscopy (XPS) characterization was performed using ESCALAB XI^+^ (Thermo Fisher Scientific) with Al Kα (1486.6 eV) as the X-ray source. Fourier-transform infrared spectroscopy (FTIR) was conducted using an FTIR spectrometer (PerkinElmer). X-ray diffraction (XRD) profiles were obtained using SmartLab 9 kW instrument (Rigaku) with a Cu-Kα radiation. Raman spectra were acquired using ALPHA 300R (WiTec) equipped with a 532 nm excitation laser and a spot size of 1 µm. Gaseous hydrogen product was quantitatively measured using a gas chromatography (GC, Shimadzu GC-2010 plus). Differential scanning calorimetry (DSC) was carried out using DSC 200 (Netzsch) from − 80 to 50 °C with a heating rate of 5 °C min^−1^ under the protection of N_2_ gas. Mechanical properties of hydrogel were characterized using the 3382 Series Universal Testing Machine (INSTRON).

## Results and Discussion

### Cationic Express Pathways in Hydrogel

The synthesis details of various hydrogel electrolytes are presented in Fig. S1 and Table S1. Both as-prepared PAPTMA and PAM hydrogels possessed approximately 63 wt% water (Fig. S2). Remarkably, distinct from the conventional PAM polymer, the PAPTMA polymer has long –N^+^R_3_ branches (Fig. 1b), which results in the greatly increased interfacial surface area of PAPTMA [29]. The prepared transparent PAPTMA hydrogel can be cut into arbitrary shapes for further characterizations (Fig. S3a), and the freeze-dried PAPTMA exhibited a continuous hierarchical porous structure (Fig. S3b). Fourier-transform infrared (FTIR) spectrum analysis of both PAM and PAPTMA hydrogels (Fig. 1c) revealed the characteristic signals at 3346 and 1651 cm^−1^, corresponding to the stretching vibration of N–H and C=O, respectively. Notably, the presence of –N^+^R_3_ groups (1480 cm^−1^) was detected in the PAPTMA [30, 31], which was further confirmed by XPS characterization (Figs. 1d and S4). Moreover, PAPTMA hydrogels exhibited good flexibility under twisting and stretching tests (Fig. 1e and Movie S1). Specifically, it demonstrated a tensile strength of 96 kPa at a strain of 680% (Fig. 1f), significantly higher than that of PAM hydrogels (48 kPa at 450%). This enhanced mechanical strength, which is ascribed to the elongated branch chain-induced entanglement and interspersion among PAPTMA networks [32], would allow for the toleration of large deformation in flexible batteries.

**Fig. 2:**
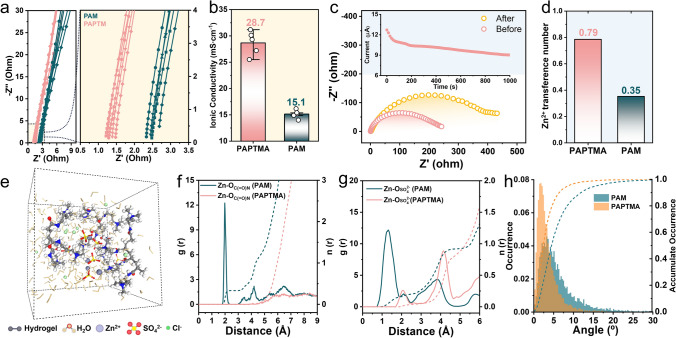
Ionic conductivity and Zn^2+^ transport of hydrogels. **a** EIS profiles and **b** the corresponding ionic conductivities of PAM and PAPTMA hydrogels. **c** EIS spectra of PAPTMA symmetric cells before and after polarization, and **d** the corresponding Zn^2+^ transference numbers. **e** Molecular configuration of PAPTMA hydrogel by molecular dynamic simulations. **f** Radial distribution functions for Zn^2+^–O (amide group) collected in PAPTMA and PAM. **g** Radial distribution functions for Zn^2+^–O (SO_4_^2−^ anions) collected in PAPTMA and PAM. **h** Statistical analysis of orientation-dependent Zn^2+^ migration

**Fig. 3:**
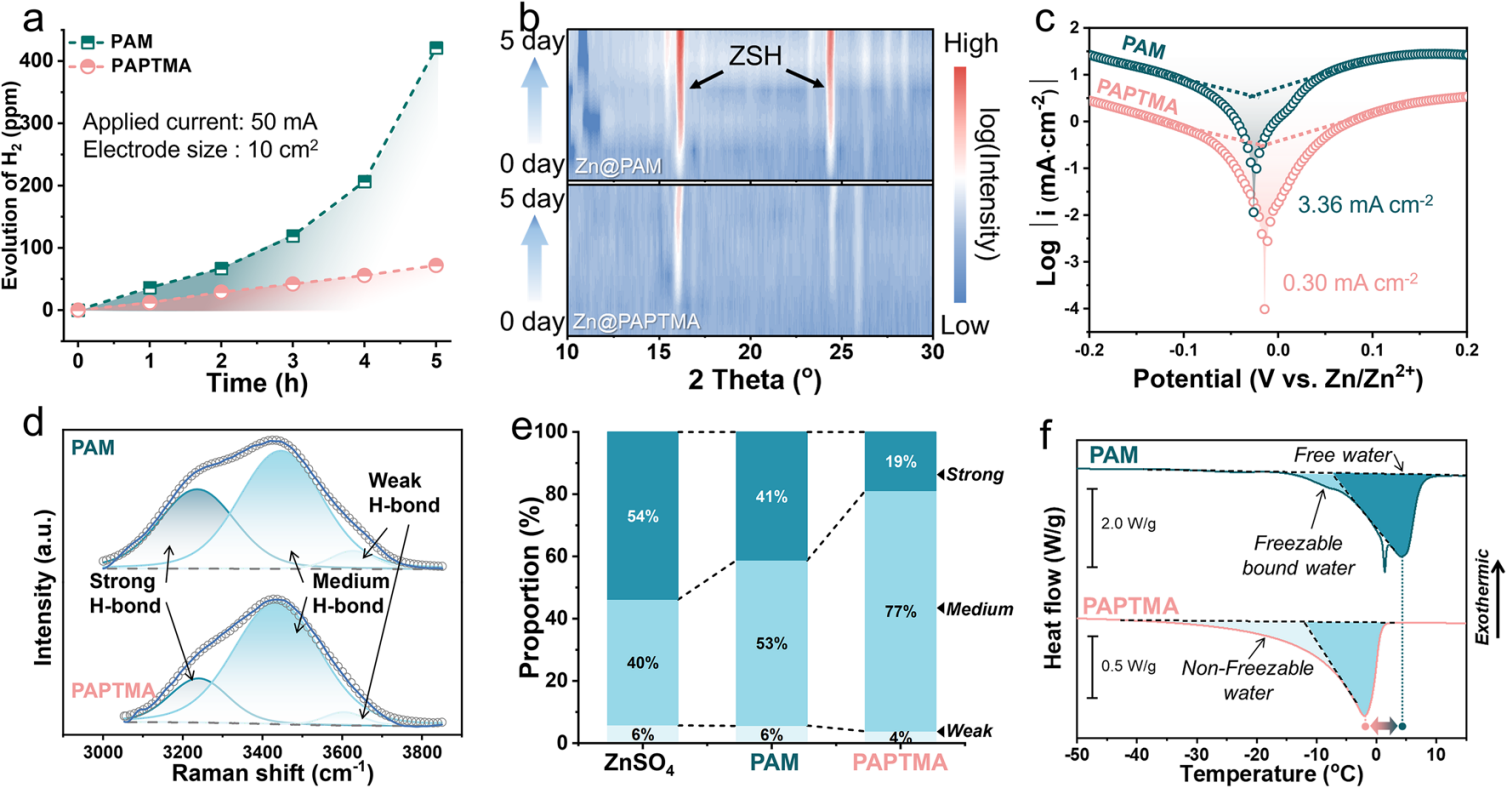
Electrochemical stability of hydrogels. **a** Time-dependent H_2_ generation using various hydrogels. **b** Ex situ XRD contours of hydrogels covered zinc foils with increasing period. **c** Tafel plots of the corrosion of zinc foil in different hydrogels. **d** Raman spectra of hydrogen bonds, and **e** the corresponding proportions of strong, medium, and weak hydrogen bonds in hydrogels. **f** DSC curves of PAPTMA and PAM hydrogel at a heating rate of 5 °C min^−1^

The ionic conductivity and cation transport capability were evaluated using the electrochemical impedance spectroscopy (EIS) technique. PAPTMA exhibited an average ionic conductivity of 28.7 mS cm^−1^ (Fig. 2a, b), which was nearly twice as high as PAM (15.1 mS cm^−1^). Moreover, assessed by the Bruce–Vincent method [33], PAPTMA showed a $t_{{{\text{Zn}}^{2 + } }}$ of 0.79 (Fig. 2c, d), surpassing the value of PAM $\left( {t_{{{\text{Zn}}^{2 + } }} = 0.36} \right)$ (Fig. S5 and Table S2). These results demonstrated the superior conducting properties of PAPTMA. To dive into the microscale mechanism of Zn^2+^ migration in hydrogels, detailed ab initio molecular dynamics (AIMD) simulations were performed using the corresponding models (Figs. 2e and S6a). The mean square displacement (MSD) results (Fig. S6b) confirmed that Zn^2+^ in PAPTMA had significantly enhanced mobility compared to that in PAM. The analysis of radial distribution functions (RDFs) revealed that the average distance between Zn^2+^ and the O_C(=O)N_ (O in amide groups) in PAM polymeric chain was as low as 1.98 Å (Fig. 2f), suggesting a hopping-mode tortuous Zn^2+^ migration in the polyanionic hydrogels [34, 35]. Conversely, the distance of Zn–O_C(=O)N_ in PAPTMA was larger than 3.84 Å (Fig. 2f) due to the ionic repulsion caused by the long cationic –N^+^R_3_ groups, indicating a distinct Zn^2+^ migration mechanism from the hopping model [36]. Regarding the SO_4_^2−^ counterions in hydrogel, the bond length of ${\text{Zn}}{-}{\text{O}}_{{{\text{SO}}_{4}^{2 - } }}$ (O in SO_4_^2−^) in PAPTMA (2.08 Å) was much longer than that in PAM (1.32 Å, Fig. 2g), which was attributed to the electrostatic attraction-induced immobilization of SO_4_^2−^ by the cationic –N^+^R_3_ groups [37]. Furthermore, the salvation structure of Zn^2+^ in PAM displayed a smaller minimum Zn–O_H2O_ distance (1.36 Å) and larger coordination number (3.88) compared to those in PAPTMA (1.76 Å and 3.05, Fig. S6c), demonstrating the weakened interaction between Zn^2+^ and H_2_O molecules in PAPTMA. This desolvation of Zn^2+^ contributed to the enhanced resistance toward parasitic HER (shown later in Fig. 3). In addition, a spatial analysis of Zn^2+^ migration reveals that Zn^2+^ transport follows a more concentrated distribution of orientation in PAPTMA (Fig. 2h), which is due to the repulsion guidance by –N^+^R_3_ branches. Taken together, we interpreted that in the PAPTMA, Zn^2+^ transport was boosted along the express pathways guided by the repulsion effect of the cationic branches (–N^+^R_3_), while SO_4_^2−^ counterions were immobilized. This leads to the simultaneous achievement of high *t* and σ.

The ionic conductivity in the hydrogel is also significantly influenced by the collision and attraction interactions with the polymeric frameworks [38]. Therefore, we have systematically tuned the monomer concentration in the initial mixture to further explore the efficacy of hydrogels. An increase in AM monomers for PAM resulted in a continued decrease of ionic conductivity (Fig. S7a, c), suggesting that ion transport was impeded by the denser polymeric matrix, while for PAPTMA, as the monomer concentration increased from 2.0 to 3.5 mol L^−1^, the ionic conductivity of the corresponding hydrogel reached a maximum of 28.7 mS cm^−1^ (Fig. S7b, c), indicating that an optimal monomer concentration (3.5 mol L^−1^) was achieved to favor the fast ion transport through cation express pathways. However, a further increase in monomer concentration led to a drop in ionic conductivity to 18.8 mS cm^−1^, which was attributed to the overcrowding of cationic polymer (Fig. S7d). Moreover, AM–APTMA copolymeric hydrogels with increasing proportions of APTMA monomers were fabricated for comparison (Table S1). When the AM–APTMA monomer ratios were set at 5:5, 3:7, and 1:9, the ionic conductivity increased by 8.6%, 15.9%, and 47.0%, respectively (Fig. S8a, b). This trend of exponential increase demonstrated the significant improvement in ionic conductivity due to the formation of a continuous cation express path, whereas the presence of AM blocks in the copolymer chain served as trapping sites that slowed down Zn^2+^ transportation (Fig. S8c). These results underscored our rational design of ionic repulsion-driven express pathways for the efficient transport of Zn^2+^.

### Restrained Water Reactivity

The electrochemical stability of hydrogels was systematically evaluated by examining the products of parasitic reactions. First, the evolution of H_2_ during the plating/stripping process in the Zn|Gel|Zn symmetric cells was quantitatively measured (Fig. S9). The concentration of H_2_ exponentially increased to 421 ppm in 5 h when using PAM, while in PAPTMA, it steadily increased to only 72 ppm (Fig. 3a), demonstrating the evidently suppressed HER in PAPTMA. Subsequently, the formation of Zn_4_(OH)_6_SO_4_·5H_2_O (ZSH) by-products resulted from the local alkaline environment triggered by HER [39] was investigated through the comprehensive compositional and morphological characterizations using XRD and SEM. By individually covering hydrogels on Zn foils for 0 to 5 days, the ex situ XRD profiles showed evident diffraction peaks of ZSH in PAM since the first day (Figs. 3b and S10a). The corresponding SEM images also exhibited the rapid growth of ZSH crystals when using PAM hydrogel (Fig. S11a). While in PAPTMA hydrogel, the formation and growth of ZSH were significantly decelerated (Figs. 3b, S10b and S11b). Moreover, Tafel plots showed a corrosion current of 0.30 mA cm^−2^ for PAPTMA (Fig. 3c), which is significantly lower than that of PAM (3.36 mA cm^−2^). Linear sweep voltammetry (LSV) tests using Na_2_SO_4_-involved hydrogel electrolyte also substantiated the suppressed HER in PAPTMA (Fig. S12). These electrochemical characterization results validated the drastically enhanced electrochemical stability of the Zn/PAPTMA interface.

The state of water plays an essential role in the performance of hydrogels. Therefore, Raman spectroscopy was applied to analyze the O–H stretching vibration mode of H_2_O, which showed characteristic peaks at 3000 ~ 3800 cm^−1^ (Figs. S13a and 3d) [40]. The component at 3250 cm^−1^ was ascribed to the strong hydrogen bond (H-bond) among the ordered tetrahedral configuration of H_2_O molecules (Fig. S14a), while the component at 3480 cm^−1^ was associated with the elongation or distortion of H-bond, representing medium H-bond in disordered H_2_O (Fig. S14b). Finally, the component at 3620 cm^−1^ was attributed to the weak H-bond of H_2_O molecules, which was due to the close interaction with the hydrophilic functional groups of the polymer (Fig. S14c) [41]. When compared to the liquid ZnSO_4_ electrolyte (Fig. S13b), the proportion of strong H-bond in PAM decreased from 54% to 41% (Fig. 3e), indicating the interruption of H-bond by the amide groups on PAM polymer, while in the PAPTMA, the proportion of strong H-bond further reduced to 19%. We interpret that, besides the presence of super hydrophilic –N^+^R_3_ groups on PAPTMA, this decline in the proportion of strong H-bond was also ascribed to the significantly increased specific surface area of PAPTMA compared to PAM [29]. These combined effects jointly led to the conversion of bulk-phase H_2_O into interfacial H_2_O molecule (92% increase in the proportion of medium H-bond, Fig. S14d).

DSC was further employed to determine the state of water in the hydrogels. During the heating scan of hydrogels, PAM exhibited a higher melting peak at 4 °C, while the endothermic peak of PAPTMA shifted to a lower temperature (Fig. 3f), indicating a decrease in bulk-phase H_2_O due to the strengthened interaction between H_2_O molecules and polymer. The significantly reduced melting enthalpy of PAPTMA (94 J g^1^) compared to PAM (231 J g^−1^) indicated the existence of non-freezable water in the PAPTMA hydrogel due to the enhanced intermolecular H-bond between polymer and water. Moreover, the completely dried PAPTMA exhibited a 10 times faster adsorption of ambient water (27 wt%) than that of the dried PAM (2.8 wt%) (Fig. S15). All these results demonstrated the extensively enhanced correlation between PAPTMA polymer and H_2_O molecules, thus contributing to the superior ionic conductivity under the lean-water state (Fig. S16). Therefore, the electrochemical stability of PAPTMA hydrogels was revealed, which was ascribed to the tuning of water state through the rational design of long cationic branches.

**Fig. 4:**
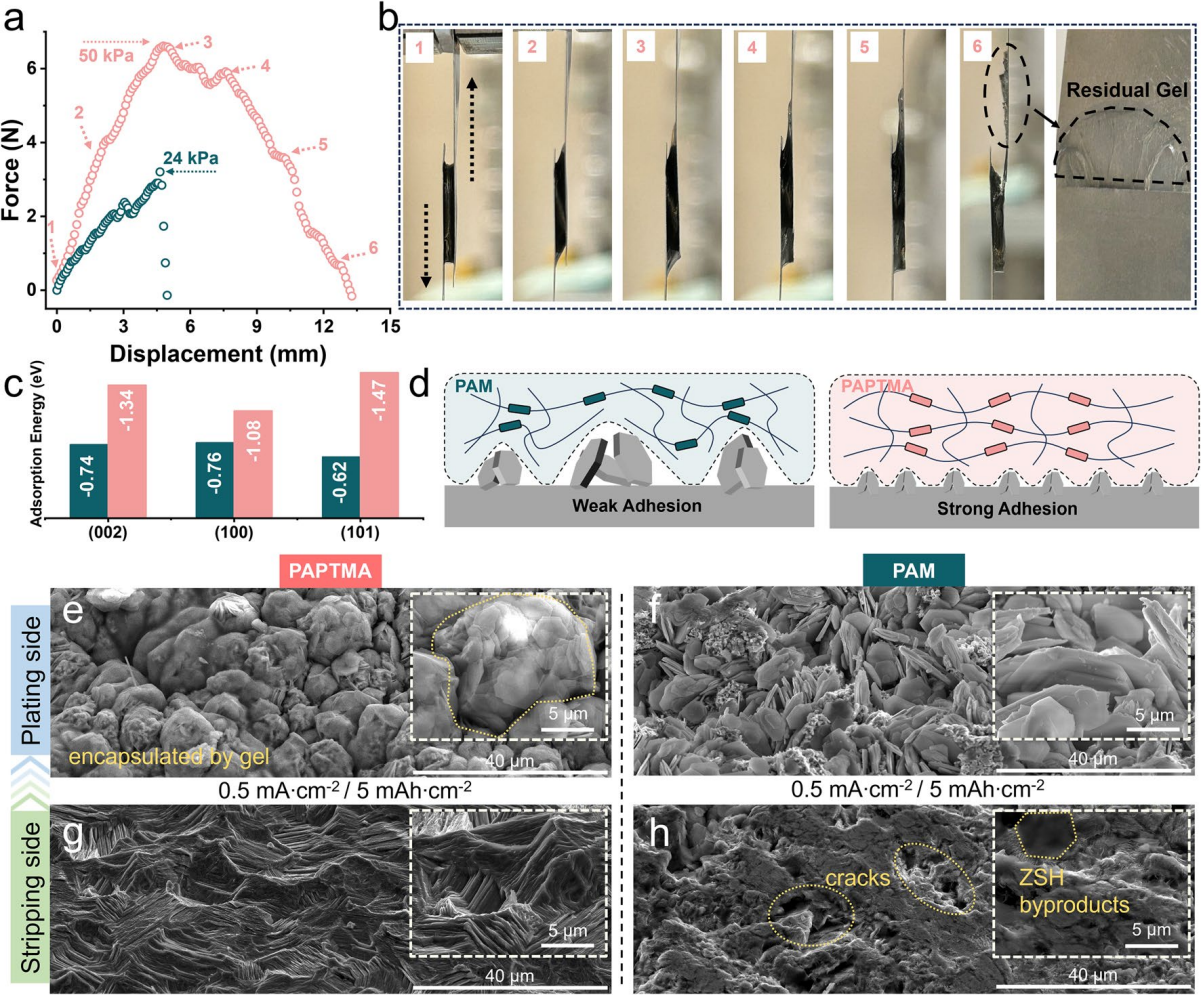
Characterization of the adhesion properties of hydrogels. **a** Shearing test of different hydrogels. **b** The corresponding optical images of stretched PAPTMA during the shearing test. **c** Adsorption energy of monomers on various planes of Zn crystal. **d** Schematic illustration showing the zinc plating at the hydrogel/anode interface. **e–h** SEM images of zinc electrode after plating (**e**, **f**) and stripping (**g**, **h**) using different hydrogels in the Zn|Gel|Zn symmetric cells

The stable adhesion of hydrogel to the Zn electrode is also essential for the durable reversibility of batteries. Therefore, the adhesion ability of hydrogel was evaluated using the lap-shear method (Fig. S17) [42]. The adhesion strength of PAPTMA hydrogel in contact with Zn foil reached 50 kPa (Fig. 4a and Movie S2), and further displacement caused the partial fracture, leaving residues on the Zn foil (Fig. 4b). In contrast, PAM easily detached from Zn foil after reaching a maximum adhesion strength of 24 kPa (Fig. S18). The superior adhesion strength of PAPTMA was partially ascribed to the low content of free-state water, which prevented the formation of lubrication layer between hydrogel and Zn foil [26, 43]. Additionally, the density functional theory (DFT) simulation proved the stronger adsorption energy between APTMA monomer and the various crystal planes of Zn (Figs. 4c and S19), contributing to the strong intrinsic adhesion.

### Mechanical Suppression of Zn Dendrites

Under the robust adhesion between PAPTMA and Zn foil, the growth of Zn dendrites could be mechanically suppressed (Fig. 4d). To further elucidate this phenomenon, in situ optical microscopy was employed to monitor the Zn plating. Remarkably, even under a high current density of 10 mA cm^−2^, Zn deposits remained flat and homogenous on the PAPTMA covered electrode, which was in stark contrast to the irregular, protuberant cluster formation observed in the PAM-based system (Fig. S20). Moreover, systematic electrochemical and microscopic morphological characterizations were implemented to gain insight into the Zn stripping/plating behavior. The chronopotentiometry (CA) tests were carried out in Zn|Gel|Zn symmetric cells. In PAPTMA, the current profile exhibited a solution diffusion characteristic (three-dimensional diffusion) with a relatively stable current after 200 s (Fig. S20), indicating the homogeneous deposition of Zn^2+^, while in PAM, the current kept increasing for over 800 s, suggesting a surface diffusion characteristic (two-dimensional diffusion) of Zn^2+^, which could lead to the growth of dendritic Zn [44, 45]. Moreover, the Zn nucleation overpotential in PAPTMA (23 mV) was much lower than that in PAM (76 mV) (Fig. S22), indicating the tight contact-induced homogeneous nucleation in PAPTMA. SEM images showed that uniform Zn flakes in the size of 3 ~ 5 μm were formed on the plating side when using PAPTMA, which were tightly encapsulated in the polymeric networks (Figs. 4e and S23). In contrast, large Zn flakes (5 ~ 15 μm) without being surrounded by polymers were grown on PAM-covered Zn foil (Fig. 4f). On the stripping side, a mixture of uneven pits and hexagonal ZSH by-products was observed when using PAM (Fig. 4h). Conversely, a clean surface without pits and ZSH by-products was observed using PAPTMA (Fig. 4g), which was owing to the uniform lattice-oriented stripping occurring at the tightly contacted PAPTMA/Zn interface [46, 47]. These remarkable distinctions substantiate the effectiveness of highly adhesive hydrogel in mechanically suppressing dendritic Zn for the reversible battery operation [48].

### Cell Performance

**Fig. 5:**
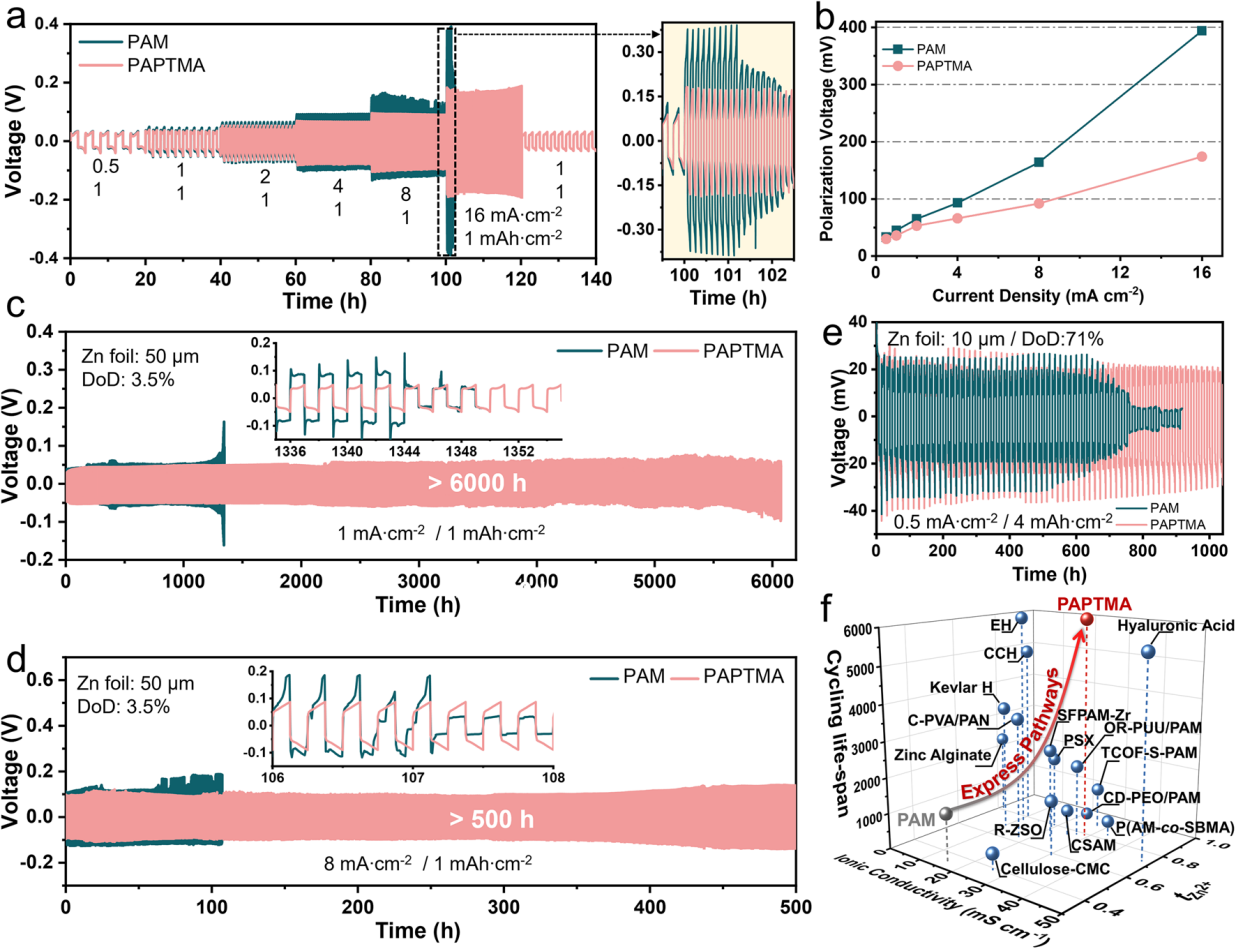
Cycling stability of symmetric cells. **a** Rate performance and **b** corresponding polarization voltage of Zn|Gel|Zn symmetric cells with the increasing current density from 0.5 to 16 mA cm^−2^. **c**–**e** Galvanostatic cycling performances of Zn‖Zn symmetric cells at **c** 1 mA cm^−2^, **d** 8 mA cm^−2^, and **e** 0.5 mA cm^−2^ (71% depth of discharge).** f** Comparison of *σ*, *t* and cycling lifespan of PAPTMA with recently reported hydrogel electrolytes

Subsequently, Zn|Gel|Cu asymmetric cells were assembled to evaluate the cycling reversibility concerning the application of PAPTMA hydrogel electrolyte. In PAPTMA, a stable operation with an average Coulombic efficiency (CE) of 99.4% was maintained over 700 cycles (Fig. S24), whereas the PAM-based cell failed within 400 cycles. The Adams method was also applied to estimate CE [49], and PAPTMA showed a superior CE of 98.7%, appreciably outperforming PAM (93.0%, Fig. S25). When integrated into Zn|Gel|Zn symmetric cells, PAPTMA cells maintained a superior rate capability under the increasing current density to 16 mA cm^−2^ (Fig. 5a), while PAM-based cells exhibited fluctuations at 8 mA cm^−2^. The apparently low overpotentials of PAPTMA indicated its rapid kinetics of Zn plating/stripping (Fig. 5b). Particularly, long-term galvanostatic cycling of PAPTMA cells achieved 6060, 1240, and 500 h under 1, 4, and 8 mA cm^−2^, respectively (Figs. 5c, d and S26), while PAM cells failed much earlier. Even at the high depth of discharge (DoD) of 71%, PAPTMA cell stably cycled for over 1000 h (Fig. 5e). Such excellent cycling stability of PAPTMA, resulted from the balance of ionic conductivity, Zn^2+^ transport, and electrochemical stability, outperformed most recently reported hydrogel electrolytes (Fig. 5f and Table S3).

**Fig. 6:**
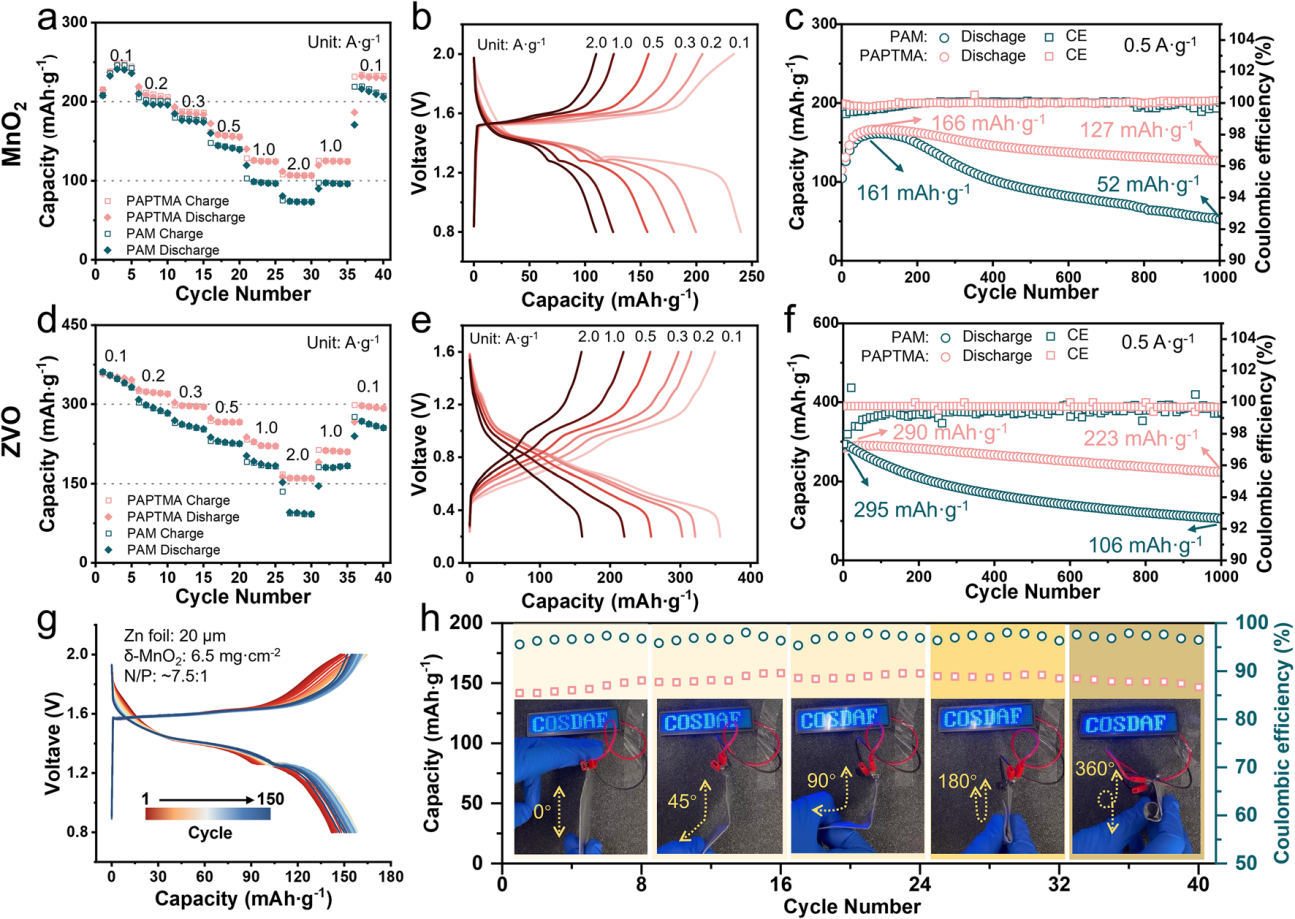
Electrochemical performance of full batteries. **a**, **d** Rate performance, **b**, **e** charge/discharge curves, and **c**, **f** cycling performance of full batteries using **a**–**c** Mn-based cathode and **d**–**f** Zn_0.25_V_2_O_5_ cathode. **g** Voltage profiles of 4 × 4 cm^2^ pouch cells at different cycles. **h** Cycling performance of a punch cell under different bending angles (Insets show the bend pouch battery that lights LED lamp beads)

The practical efficacy of PAPTMA hydrogel in ZIBs was further examined by assembling full cells using both *δ*-MnO_2_ and Zn_0.25_V_2_O_5_ (ZVO) as cathode materials. The cyclic voltammetry (CV) curves of Zn|Gel|MnO_2_ cells showed a smaller gap between the redox reactions in PAPTMA hydrogel when compared to PAM (Fig. S27), indicating fast reaction kinetics of PAPTMA. For both δ-MnO_2_ and ZVO cathodes, PAPTMA cells (Fig. 6a, b) showed higher specific capacity than those of PAM-based cells (Fig. 6d, e) at increasing current density, demonstrating the promoted kinetics of PAPTMA hydrogel. Particularly, after 1000 cycles at a current density of 0.5 A g^−1^, PAPTMA cells still delivered 76.5% (127 mAh g^−1^) and 76.9% (230 mAh g^−1^) capacity retention integrating with δ-MnO_2_ and ZVO cathodes, respectively (Fig. 6c, f), whereas PAM cells decayed quickly in the first 300 cycles. Ex situ EIS analysis demonstrated distinct interfacial stability characteristics between PAPTMA and PAM-based cells during the initial 100 cycles. The PAPTMA cells maintained a consistent charge transfer resistance (Fig. S28a), corroborated by the preserved electrode–electrolyte interface morphology observed in SEM images (Fig. S29a, b). In contrast, PAM cells exhibited a progressive increase in charge transfer resistance (Fig. S28b), attributable to the structural deterioration of PAM as evidenced by SEM characterization (Fig. S29c, d). Furthermore, comprehensive evaluation of environmental adaptability revealed the superior performance of PAPTMA across extreme temperature conditions. The full cells incorporating PAPTMA demonstrated significantly enhanced capacity retention compared to PAM-based counterparts at both subzero (− 15 °C) and elevated (60 °C) temperatures (Figs. S30 and S31), underscoring remarkable thermal stability and environmental robustness of PAPTMA.

Encouraged by the impressive performance of PAPTMA, we proceeded to assembled 4 × 4 cm^2^ pouch cells with a relatively high δ-MnO_2_ mass loading of ~ 6.5 mg cm^−2^. The CV curves at an increasing scan rate showed a small variation of redox potential (Fig. S32), indicating the superior kinetics of PAPTMA [50]. At a current density of 0.5 A g^−1^, the PAPTMA pouch with a N/P ratio of ~ 7.5 cell delivered a consistent energy density of 206 Wh kg^−1^_(MnO2)_ (corresponding to 1.36 V and 152 mAh g^−1^ of δ-MnO_2_) for 150 cycles (Figs. 6g and S33). Notably, even under a bending test spanning from 0° to 360° (Fig. 6h), the specific capacity was well maintained, indicating the robustness of the PAPTMA/electrode interface. These results validated that the cationic PAPTMA hydrogel, integrating the ionic repulsion-driven express pathways for Zn^2+^ transport, significantly enhanced the operating stability of ZIBs in practical cells.

## Conclusions

In summary, we have designed a cationic PAPTMA hydrogel for the durable ZIBs by efficiently addressing the critical trade-offs among ionic conductivity, Zn transference number, and electrochemical stability of the conventional hydrogel electrolytes. The ionic repulsion-driven express pathways formed by –N^+^R_3_ branch chains of PAPTMA polymeric matrix contributed to the fast transport of Zn^2+^ $\left( {t_{{{\text{Zn}}^{2 + } }} = 0.79} \right)$. Moreover, the free-state water in PAPTMA hydrogel was converted to interfacial water due to the existence of long hydrophilic –N^+^R_3_ branches, thus balancing the ionic conductivity (σ = 28.7 mS cm^−1^) and water-induced parasitic reactions. Consequently, symmetric cells using PAPTMA hydrogel exhibited a stable cycling lifespan over 6000 h at 1 mA cm^−2^, and the 4 × 4 cm^2^ pouch cells showed maintained capacity (150 mAh g^−1^) under 0 ~ 360° bending. Our strategy balances the three-way trade-offs of hydrogel electrolytes by forming ionic repulsion-driven express pathways, which may make a leap forward for the development of flexible ZIBs.

## Supplementary Information

Below is the link to the electronic supplementary material.

Supplementary file1 (MP4 3747 KB)

Supplementary file2 (MP4 7611 KB)

Supplementary file3 (DOCX 7168 KB)
