# Supplementary material for: Water-Restrained Hydrogel Electrolytes with Repulsion-Driven Cationic Express Pathways for Durable Zinc-Ion Batteries
Source: Nanomicro Lett. 2025 Mar 19;17:193. doi: 10.1007/s40820-025-01704-5 (PMC11920515; doi:10.1007/s40820-025-01704-5)
Supplement: Supplementary file 3 — Supplementary file3 (DOCX 7168 KB) [file 40820_2025_1704_MOESM3_ESM.docx]

Supporting Information for

**Water-Restrained Hydrogel Electrolytes with Repulsion-Driven Cationic Express Pathways for Durable Zinc Ion Batteries**

Dewu Lin^1^, Yushuang Lin^2^, Ruihong Pan^1^, Jiapei Li^1^, Anquan Zhu^1^, Tian Zhang^1^, Kai Liu^1^, Dongyu Feng^1^, Kunlun Liu^1^, Yin Zhou^1^, Chengkai Yang^2,^*, Guo Hong^1,3,^*, and Wenjun Zhang^1,^*

^1^ Department of Materials Science and Engineering & Center of Super-Diamond and Advanced Films (COSDAF), City University of Hong Kong, Hong Kong 999077, P. R. China

^2^ College of Materials Science and Engineering, Fuzhou University, Fujian 350108, P. R. China

^3^ The Shenzhen Research Institute, City University of Hong Kong, Shenzhen 518057, P. R. China

*Corresponding authors. E-mail: [chengkai_yang@fzu.edu.cn](mailto:chengkai_yang@fzu.edu.cn) (Chengkai Yang); [guohong@cityu.edu.hk](mailto:guohong@cityu.edu.hk) (Guo Hong); [apwjzh@cityu.edu.hk](mailto:apwjzh@cityu.edu.hk) (Wenjun Zhang)

**S1 Experimental Section**

**S1. 1 Materials**

Zn foil (10~100 μm, Canrd New Energy Technology). ZnSO_4_·7H_2_O (≥99.5%); Acrylamide (≥99.9%); (3-Acrylamidopropyl) trimethyl ammonium chloride solution (75 wt.% in H_2_O); N, N’-methylenebisacrylamide (≥99.5%); Potassium persulfate (≥99.5%). Mn(Ac)_2_·4H_2_O (≥99.9%); N_2_H_4_·H_2_O (50% wt.% in H_2_O); NaClO (6-14% active chlorine basis); V_2_O_5_ (≥99.0%); Zinc acetate (≥99.0%) are purchased from Sigma-Aldrich.

**S1.2 Preparation of cathode materials**

The δ-MnO_2_ cathode material was prepared using a previously reported method. Typically, 4 mmol Mn(Ac)_2_·4H_2_O was dissolved into 40 mL DI water, followed by adding 3 mL N_2_H_4_·H_2_O (50%). The resulted solution was transferred into a Teflon-lined stainless-steel autoclave and heated at 180 ℃ for 12 hours. Afterward, a white solid was obtained after washing the product with DI water and ethanol and dried at 60 ℃ overnight. Subsequently, 0.2 g obtained white powder was dissolved into 50 mL DI water, followed by adding 10 mL of NaClO (>10%) solution. The mixture was stirred for 24 h, resulting in the formation of δ-MnO_2_. The black δ-MnO_2_ sample was finally collected by filtration and washed with DI water.

Zn_0.25_V_2_O_5_ cathode material was synthesized by the hydrothermal method as well. Typically, 4 mmol V_2_O_5_ and 2.6 mmol zinc acetate were dispersed in 100 ml mixed solvent (DI : acetone : 10%HNO_3_ = 92:6:2 in volume). The resulted solution was transferred into a Teflon-lined stainless-steel autoclave and heated at 180 ℃ for 24 hours. The dark blue product was available after thorough washing and drying.

The cathode slurry for full cell was prepared by mixing δ-MnO_2_ (or Zn_0.25_V_2_O_5_), Super P (10 wt%), and PVDF binder (10 wt%) in N-methyl-2-pyrrolidone (NMP) to form a homogeneous slurry. Then, the slurry was uniformly coated on carbon cloth using a doctor blade and dried in an oven at 60 °C for 12 h. The areal δ-MnO_2_ (or Zn_0.25_V_2_O_5_) loading was controlled around 1.0~1.5 mg cm^–2^.

**S1.3 Electrochemical Measurements**

All the symmetric and asymmetric batteries were assembled in CR2032 coin-type cells for testing. The full cell components used were as follows: a polished Zn foil (20 μm in thickness, 12 mm in diameter) as anode, a slice of hydrogel (0.5 mm in thickness and 13 mm in diameter) as electrolyte, and carbon cloth supported cathode material. In the case of punch cell, area of Zn anode, hydrogel electrolyte and cathode material (~6.5 mg·cm^-2^) were expanded to 4×4 cm^2^ with other parameters unchanged.

Galvanostatic charge/discharge profiles were recorded using a NEWARE battery-testing instrument. Other electrochemical examinations, including chronoamperometry (CA) tests with an applied voltage of 100 mV, electrochemical impedance spectroscopy (EIS) over a frequency range from 100 kHz to 100 mHz, corrosion current measurements, chronopotentiometry (CP) tests with a current density of 0.5 mA·cm^-2^, linear sweep voltammetry (LSV) with a scan rate of 1 mV·s^-1^, and cyclic voltammetry tests (CV) were implemented using ModuLab XM test system (AMETEK Inc.). The Zn^2+^ transference number (*t*$\text{Zn}^{\text{2+}}$) was determined using the Bruce-Vincent method. All symmetric cells were subjected to a 30-minute rest before the examination of *t*$\text{Zn}^{\text{2+}}$to allow the stabilization of the hydrogel/electrode interface.

The ionic conductivity of hydrogel electrolyte was determined at 25 ℃ by measuring the EIS using two stainless steel electrodes and calculated according to the following equation:

σ = L/(R×S)

where R is the resistance based on EIS measurement, L is the thickness of hydrogel (0.5 mm), and S is contact area (1.13 cm^2^) between electrode and hydrogel. Importantly, to avoid deformation of hydrogel under the pressure of fixture, two pieces of 0.5-mm thick rubber were placed between electrodes as a support.

**S2 Theoretical Calculation and Simulation**

The electrostatic potential (ESP) distributions of the AM and APTMA monomers were evaluated using Density Functional Theory (DFT) with the Gaussian 16 software package. The Becke, three-parameter, Lee-Yang-Parr (B3LYP) exchange-correlation functional was employed in conjunction with the 6-311+G(d,p) basis set. The DFT calculations in this study were conducted using the CP2K^1^ software package (version 7.1). The minimal energy structures studied in this paper were obtained by optimizing with the DZVP-MOLOPT-SR-GTH^2^ basis set, PBE^3,4^ functional, and Grimme’s D3(BJ) dispersion correction.^5^ Electronic energy calculations were also performed under this configuration. Through cp2k, the adsorption energy was computed to investigate the adsorption characteristics of AM and APTMA on Zn. The definition of adsorption energy is as follows:

△E_adb_=E_AB_-E_A_-E_B_

The term E_AB_ represents the overall energy of AM and APTMA on Zn. E_A_ represents the overall energy of AM and APTMA, while E_B_ represents the energy of Zn.

**S3 Supplementary Figures and Tables**


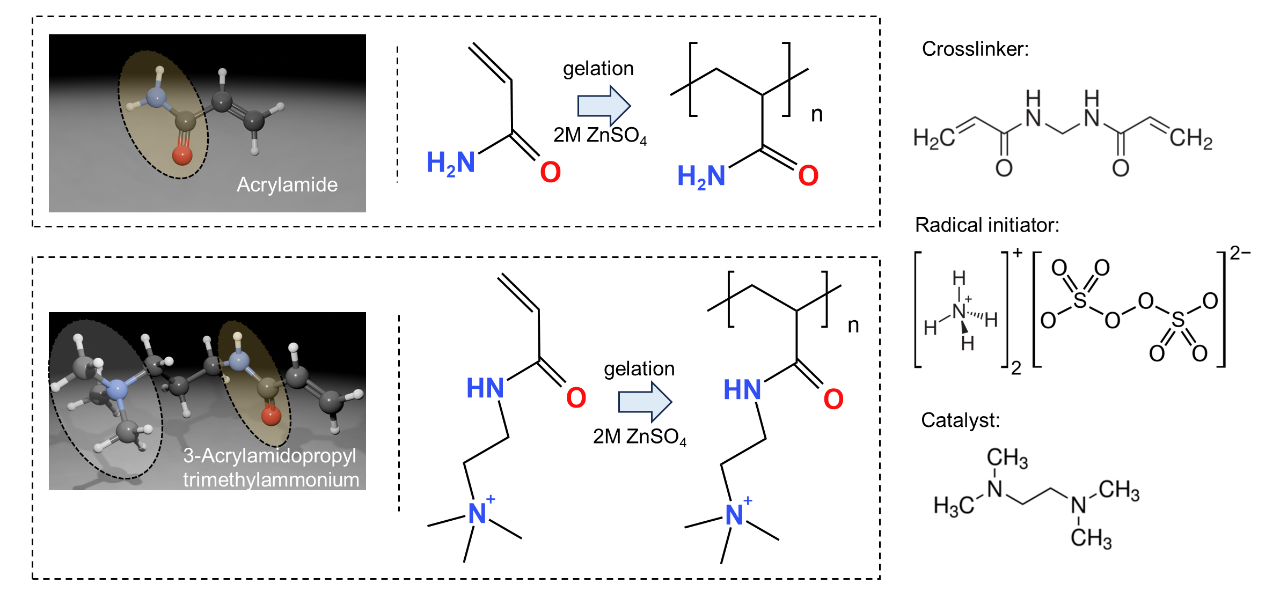


**Fig. S1** Synthesis procedure of PAM and PAPTMA hydrogels


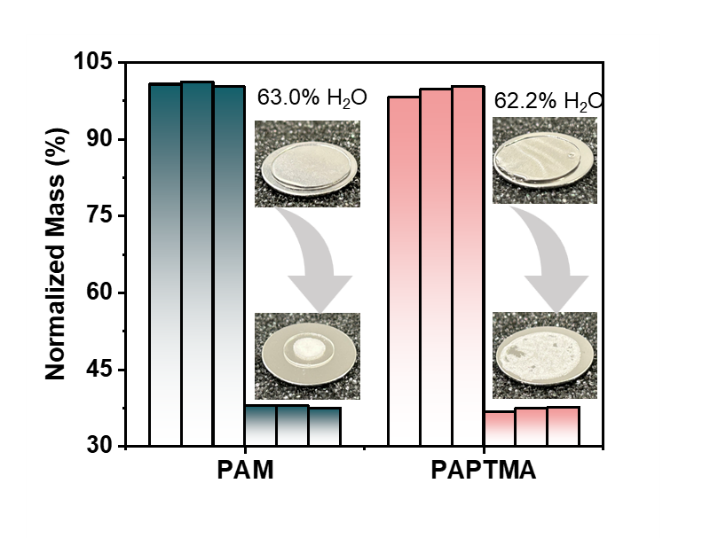


**Fig. S2** Normalized mass of PAM and PAPTMA hydrogels before and after completely drying process. Insets are corresponding optical images


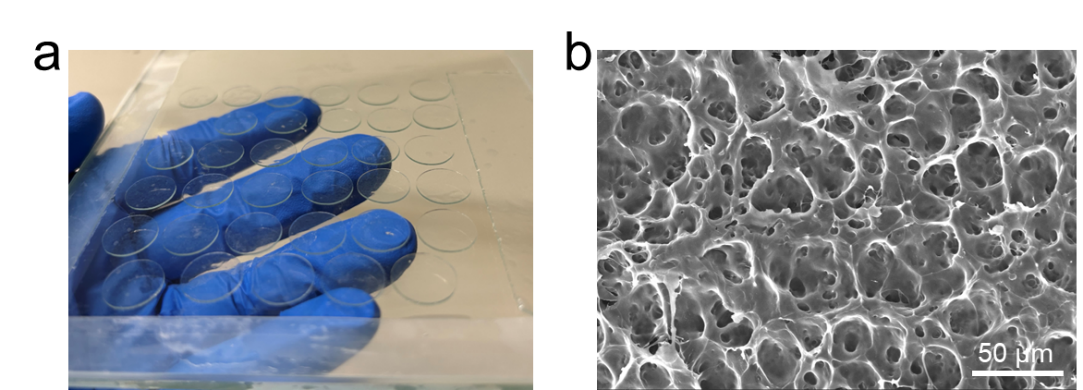


**Fig. S3** **a**) Optical and **b**) SEM image of PAPTMA cationic hydrogel


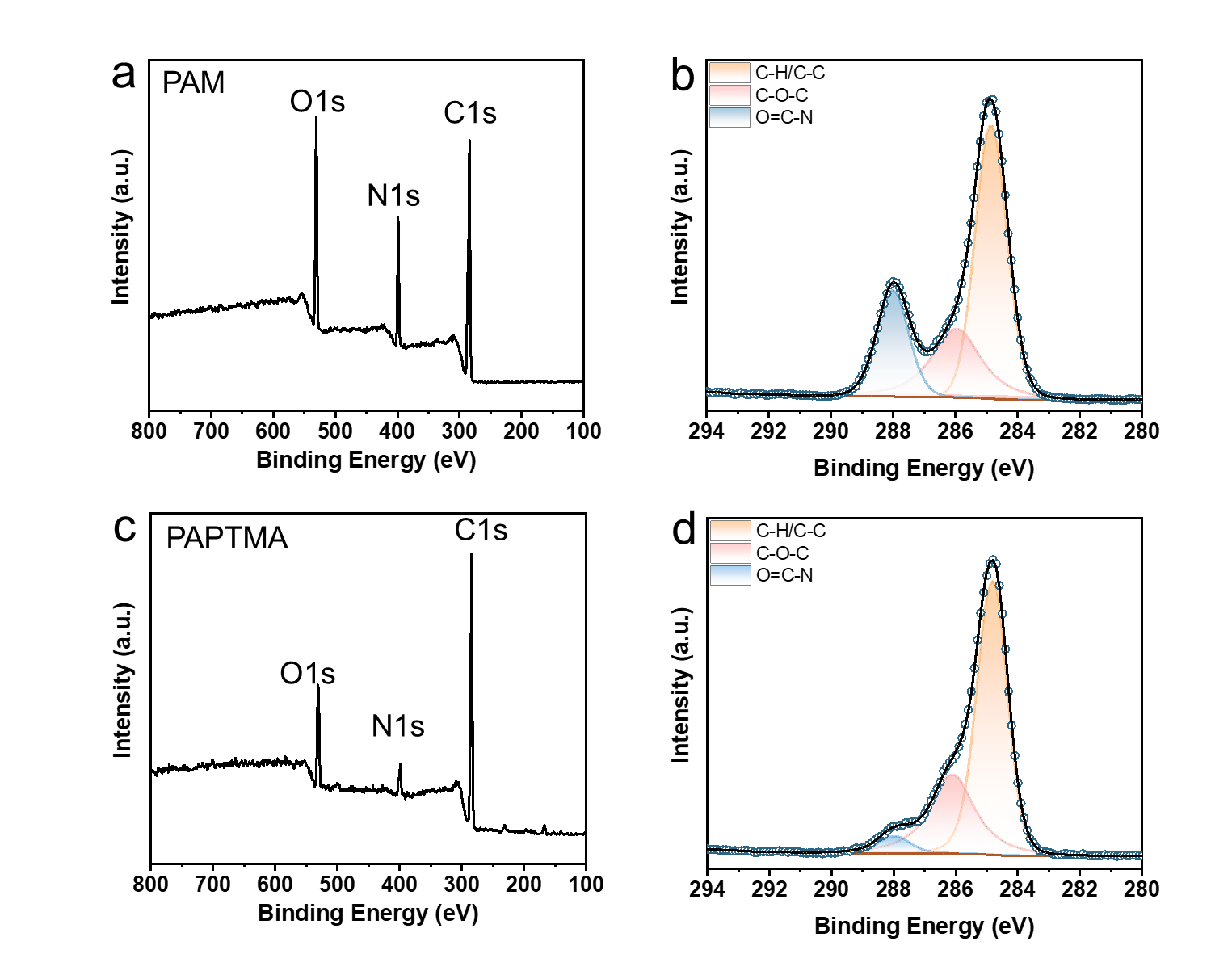


**Fig. S4** XPS characterization of PAM and PAPTMA. **a, c**) Survey and **b, d**) O1s XPS profiles of **a, b**) PAM and **c, d**) PAPTMA hydrogel


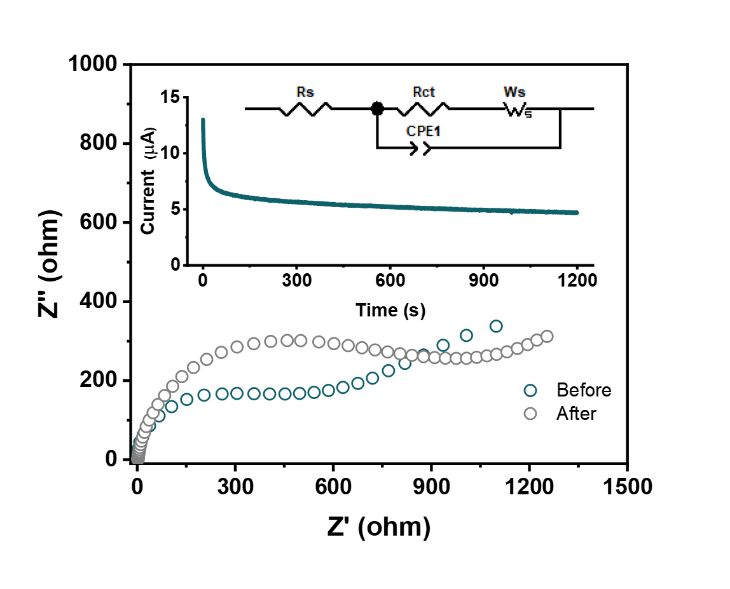


**Fig. S5** EIS profiles of Zn/PAM/Zn symmetric cell. The insets show the CA curve before and after polarization with a constant voltage of 10 mV and equivalent circuits. All the cells for transference number test were rested for 0.5 h after assembly. Fitted results are listed in Table S2


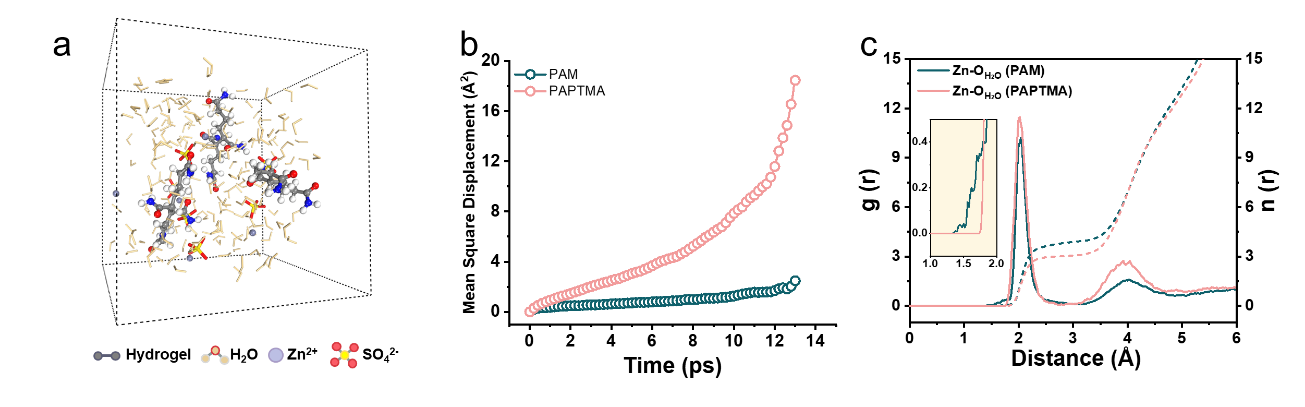


**Fig. S6** AIMD simulation of hydrogels. **a**) Molecular configuration of PAM. **b**) Mean square displacement of Zn^2+^ in PAM and PAPTMA hydrogels. **c**) Radial distribution functions for Zn-O (H_2_O) collected in PAM and PAPTMA


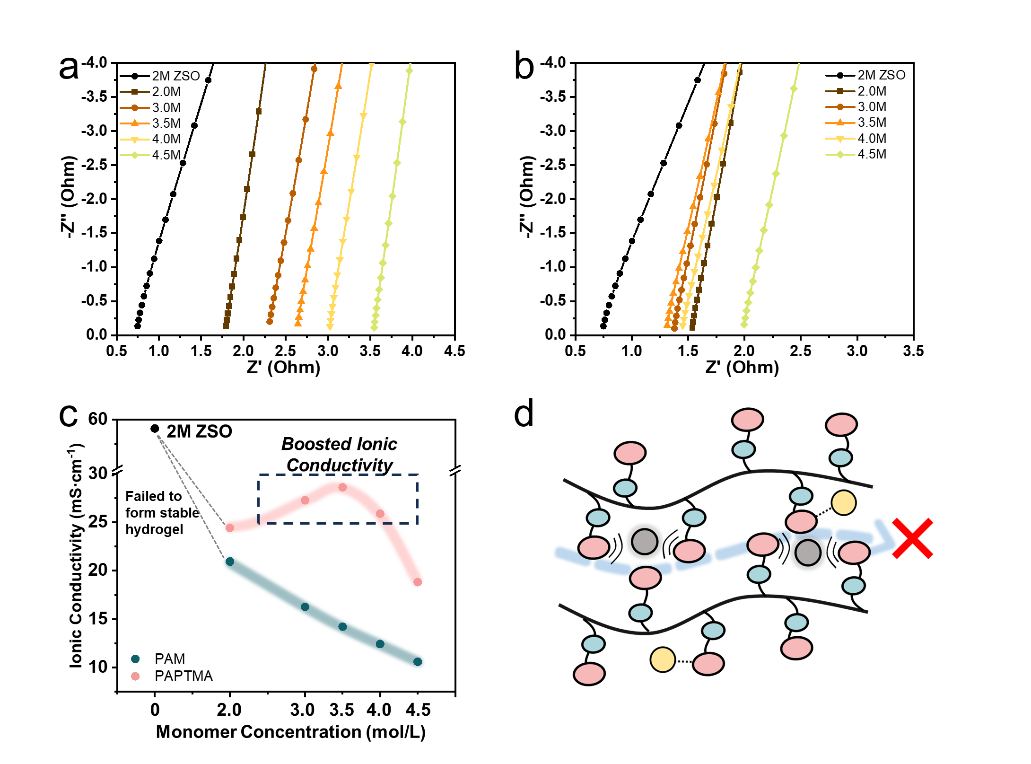


**Fig. S7** Characterization of ionic conductivity. **a, b**) EIS profiles of PAM (**a**) and PAPTMA (**b**) hydrogels with increasing monomer concentration. **c**) Summary of corresponding ionic conductivity using various concentration of monomers. **d**) Schematic illustration shows the impeded transport of Zn^2+^ in dense PAPTMA hydrogel


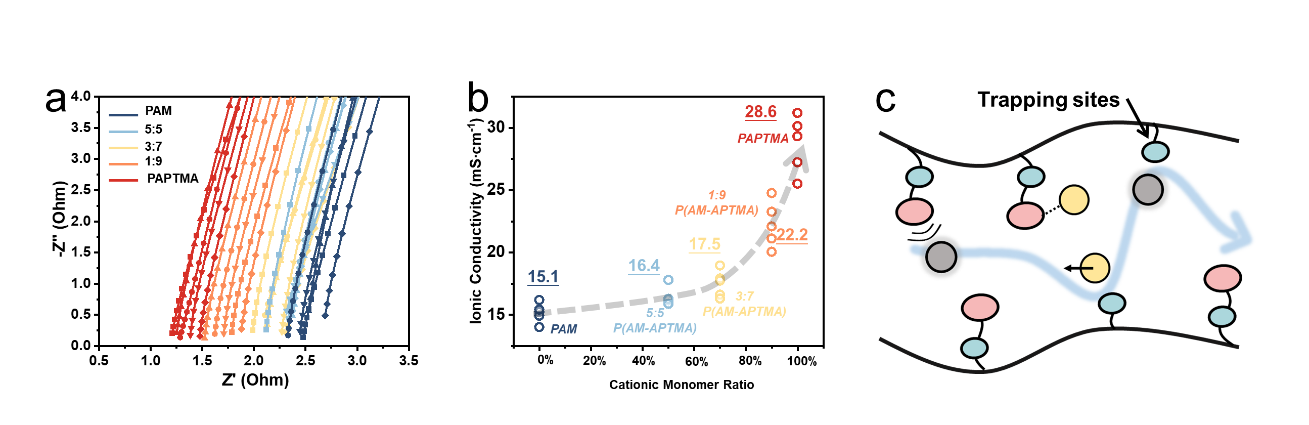


**Fig. S8** Ionic conductivity of AM-APTMA copolymer hydrogels. **a**) EIS profiles and **b**) corresponding ionic conductivity of AM-APTMA with various monomer ratio. **c**) Schematic illustration shows the “trapping site role of AM blocks in the copolymer chain


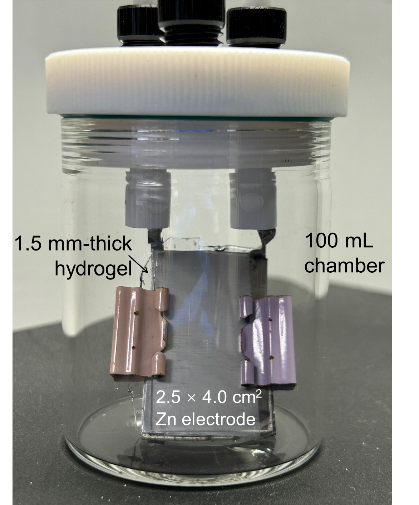


**Fig. S9** Equipment for the measurement of H_2_ evolution using different hydrogel and symmetric Zn electrodes. Applied current: 50 mA


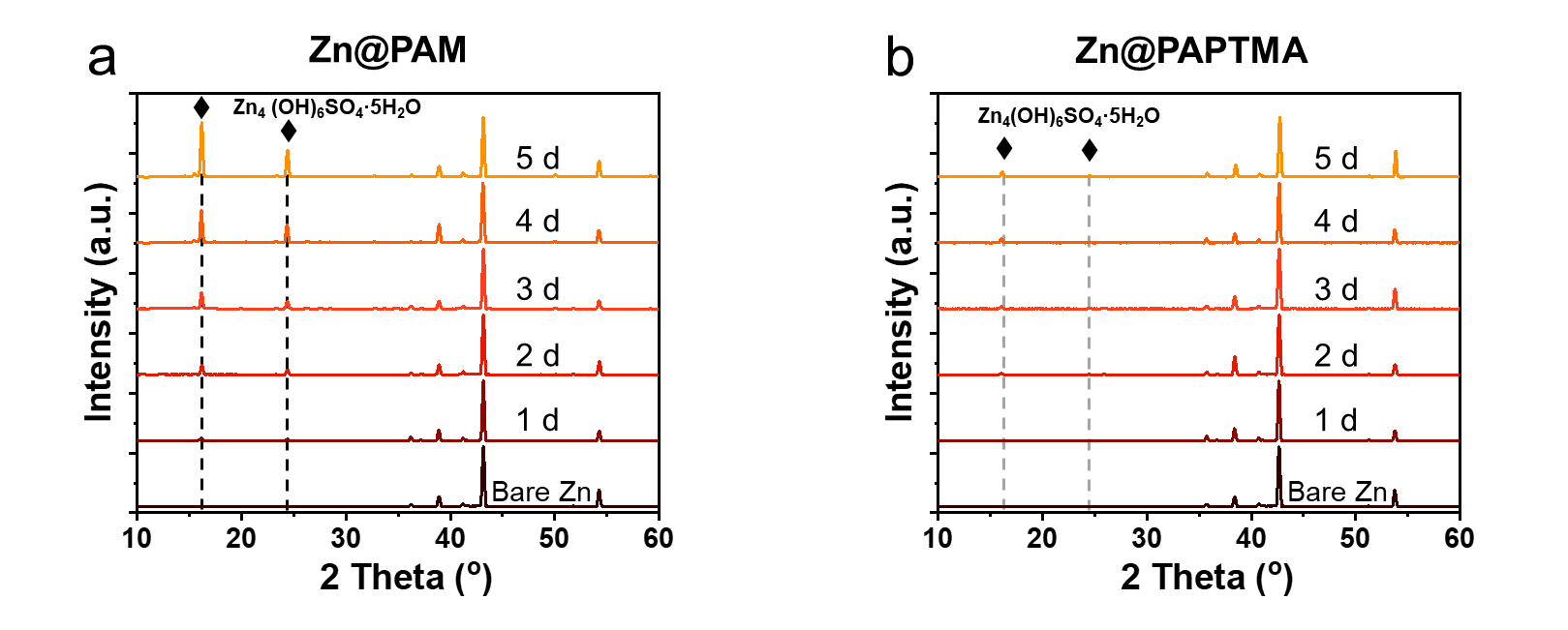


**Fig. S10** XRD profiles of Zn foils covered by PAM (**a**) and PAPTMA (**b**) for increasing duration
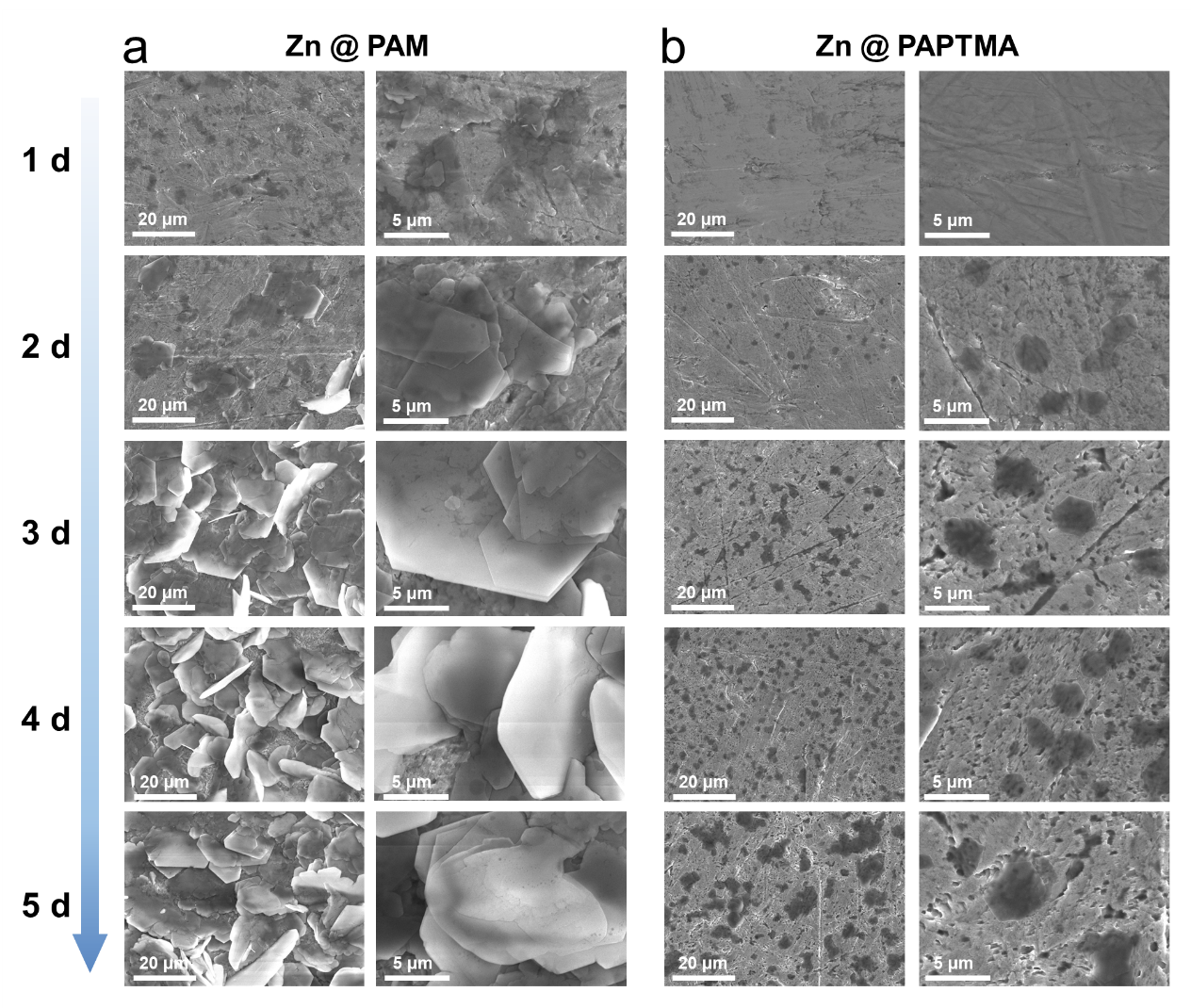


**Fig. S11.** Top-view SEM images of the Zn electrode covered by PAM (**a**) and PAPTMA (**b**) for increasing duration


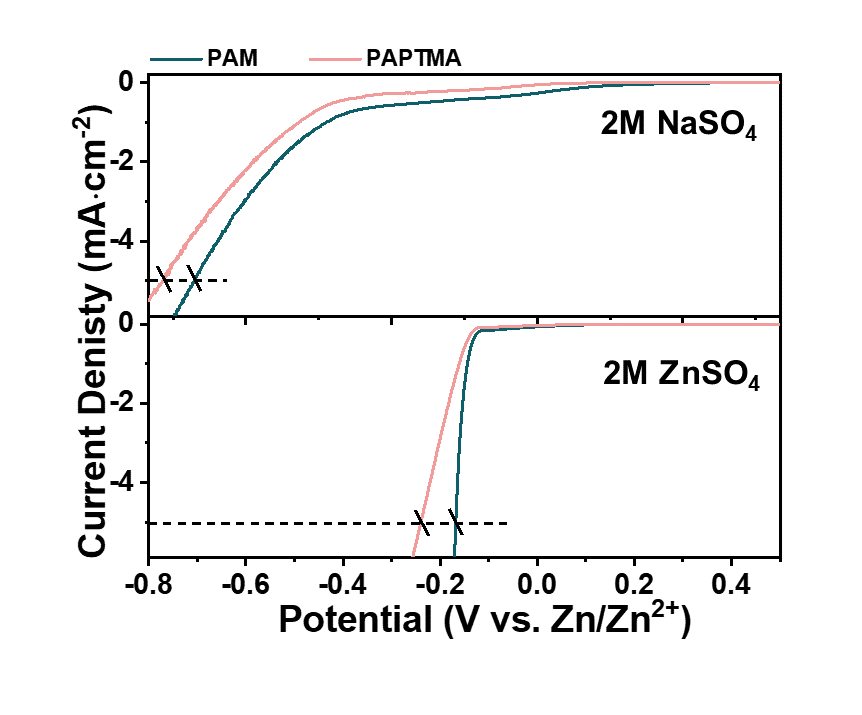


**Fig. S12** LSV curves of hydrogels using NaSO_4_ (**a**) and ZnSO_4_ (**b**) as electrolyte


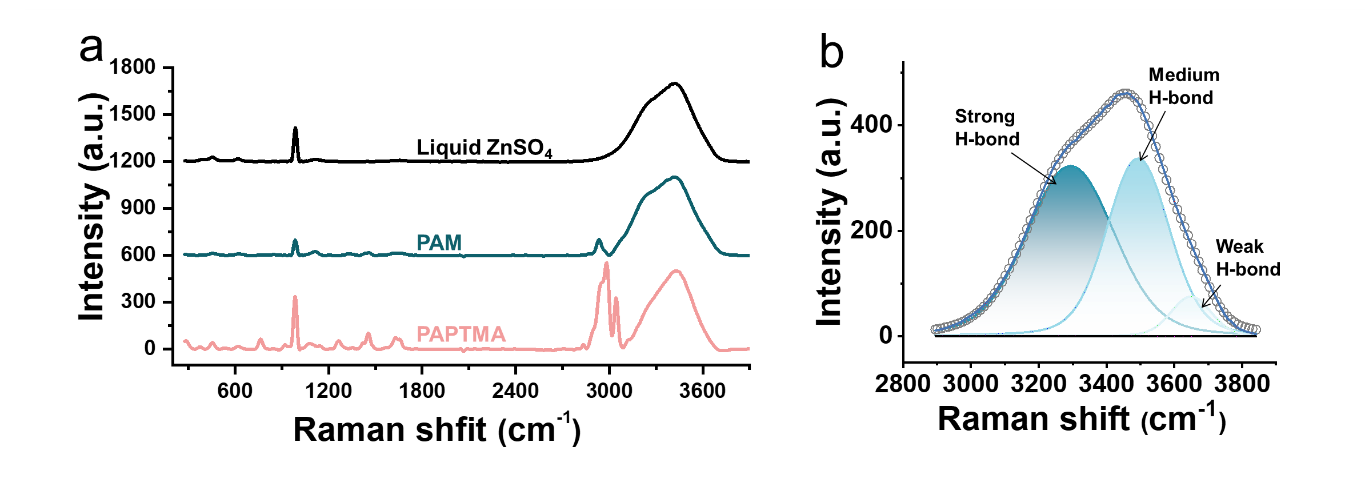


**Fig. S13** Raman characterization. **a**) Comparison of Raman spectra of hydrogels and liquid ZnSO_4_ electrolyte. **b**) Raman spectrum of O-H vibration in liquid ZnSO_4_ electrolyte


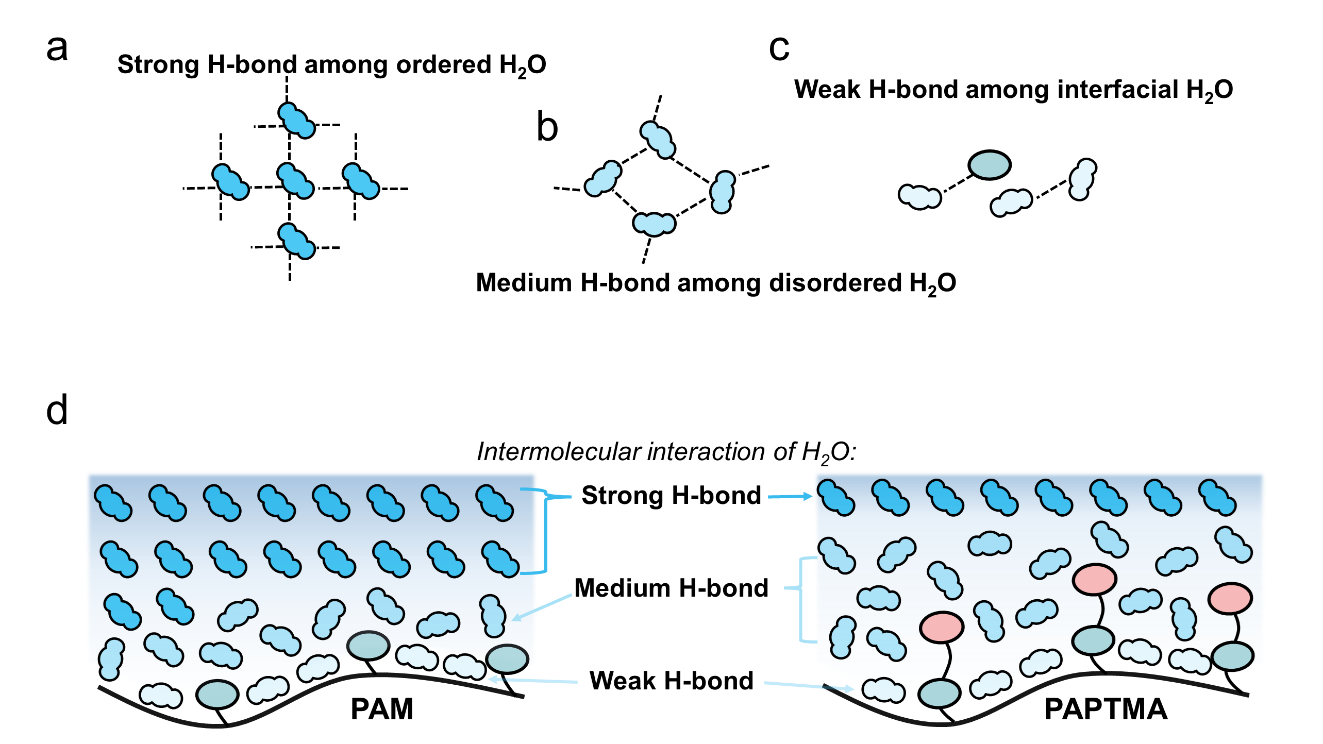


**Fig. S14** Schematic illustrations of three types of hydrogen bond in the hydrogel. **a**) Strong H-bond. **b**) Medium H-bond and **c**) Weak H-bond. **d**) Spatial distribution of H-bonds in the hydrogels


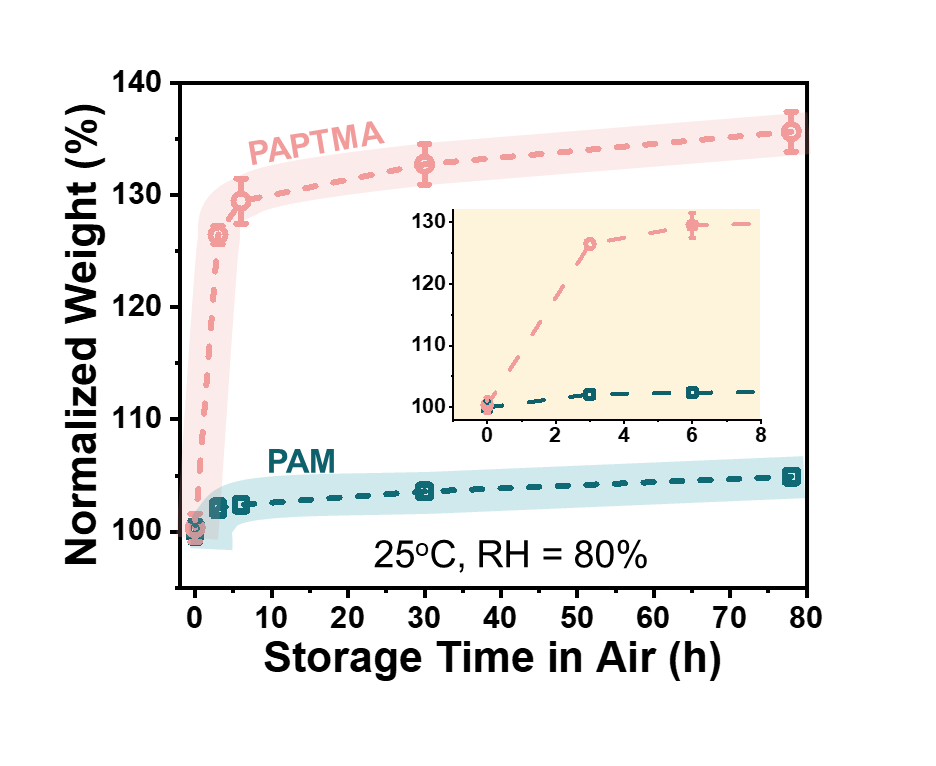


**Fig. S15** Water adsorption of dried hydrogels in ambient air


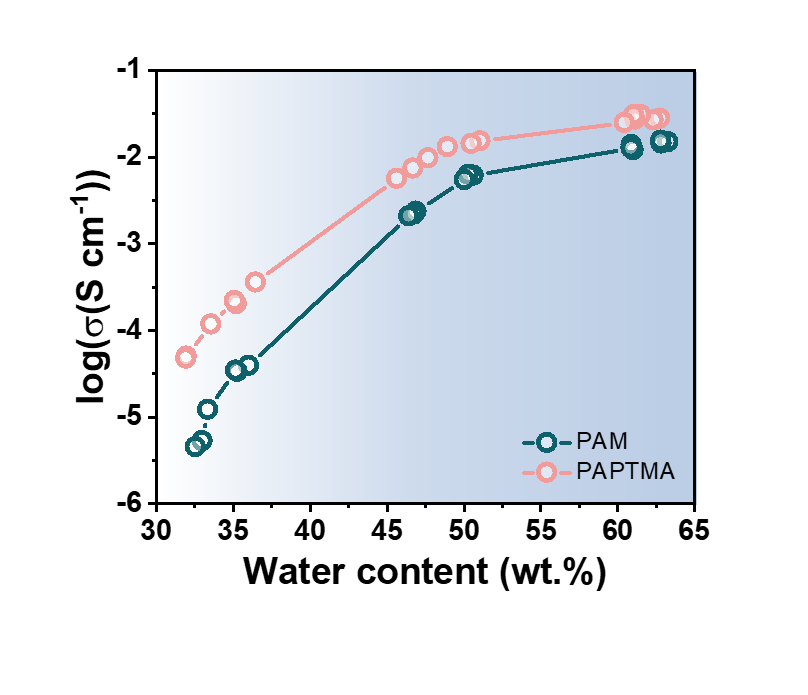


**Fig. S16** Ionic conductivity of hydrogels at reduced water content


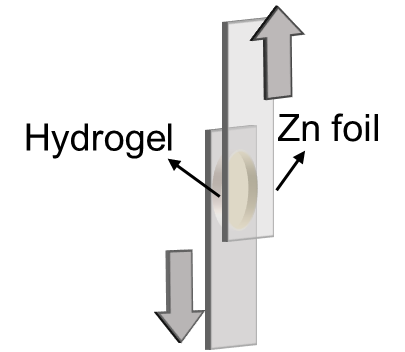


**Fig. S17** Adhesion characterization of hydrogels using lap-shear method. The thickness and diameter of cylinder-shape hydrogel was 1.5 mm and 13 mm, respectively


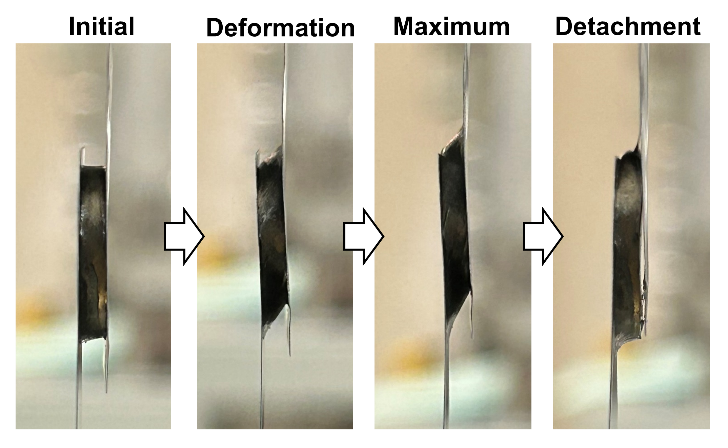


**Fig. S18** Optical images of lap-shear test of PAM hydrogel


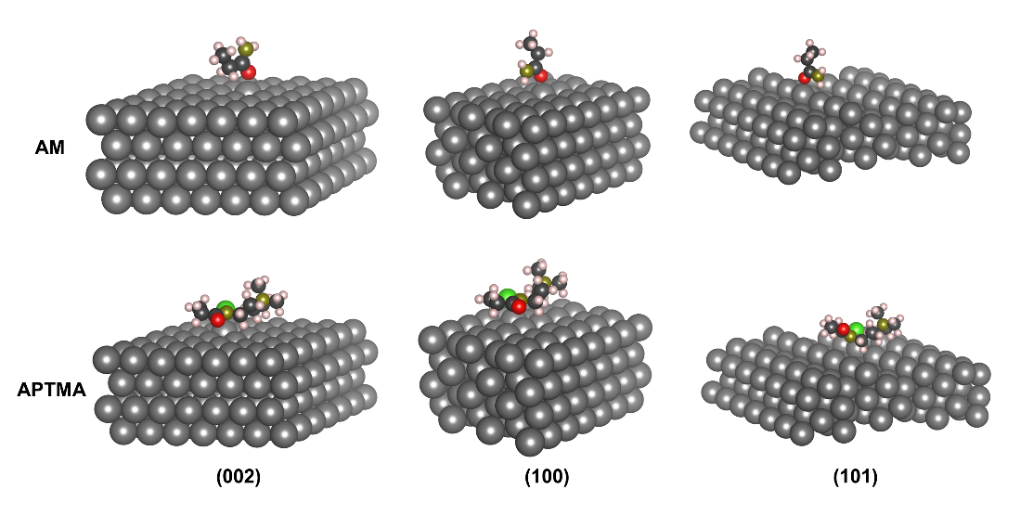


**Fig. S19** Optimized adsorption structures of AM and APTMA monomers on different Zn crystal planes


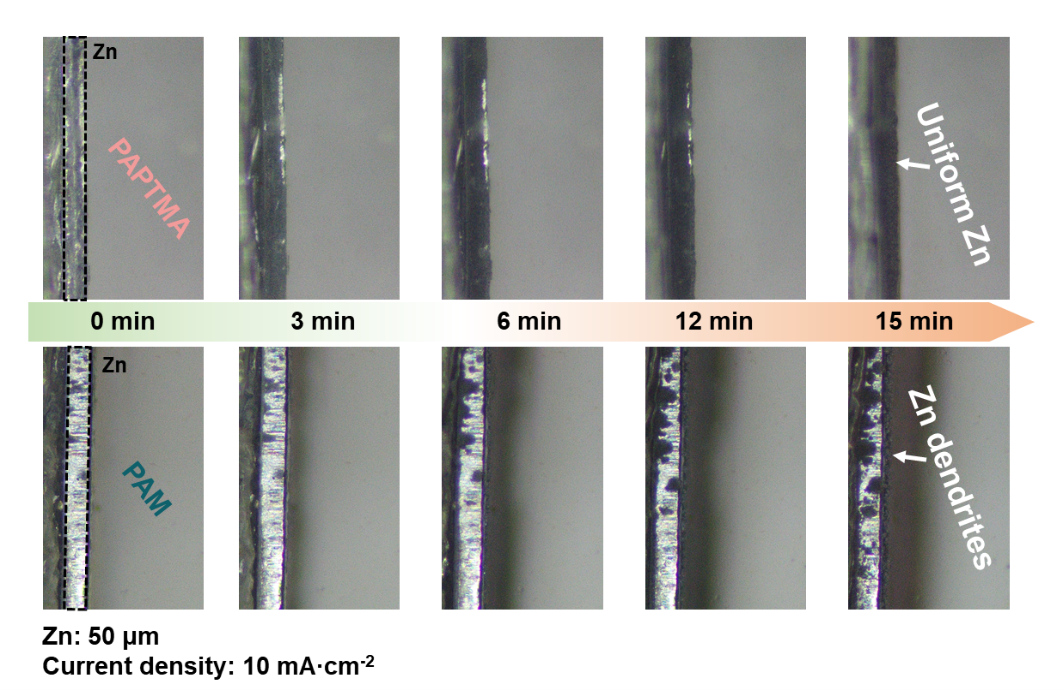


**Fig. S20** In situ optical microscopy of the Zn plating process using PAPTMA and PAM hydrogels


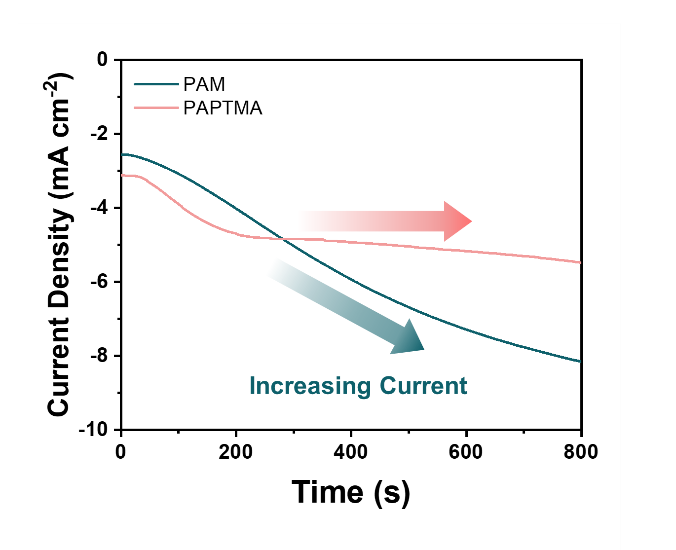


**Fig. S21** Chronopotentiometry characterization of Zn|Gel|Zn symmetric cells at an applied voltage of 100 mV


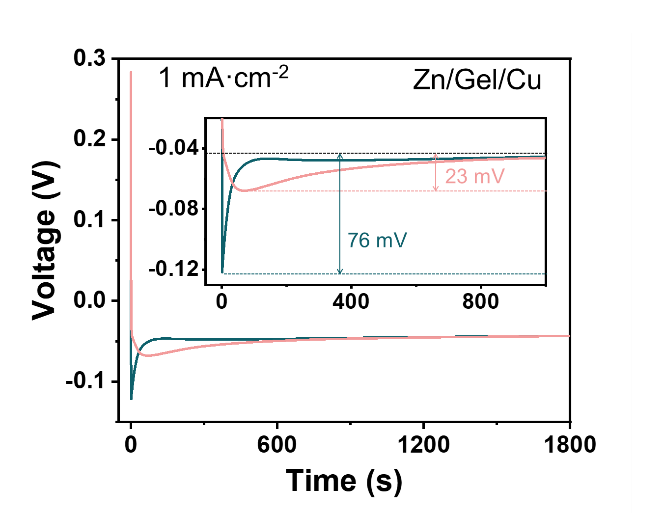


**Fig. S22** Characterization of nucleation overpotential of Zn|Gel|Cu asymmetric cells at a current density of 1 mA∙cm^-2^


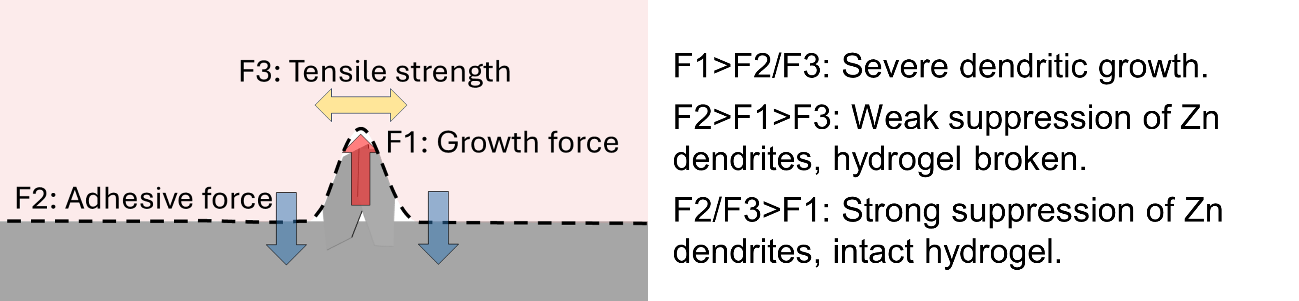


**Fig. S23** Mechanical suppression of Zn dendritic growth


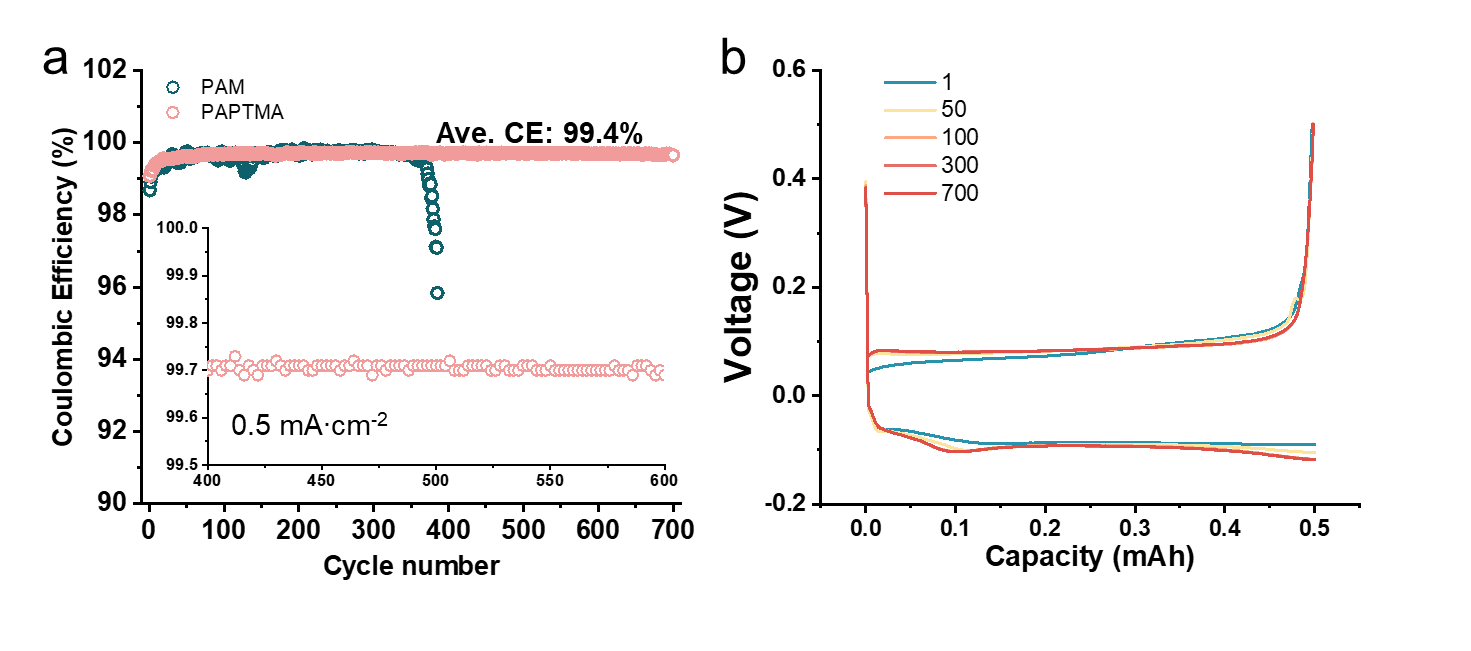


**Fig. S24** Electrochemical characterization of Zn|Gel|Cn asymmetric cells. **a**) Coulombic efficiency. **b**) Voltage profiles


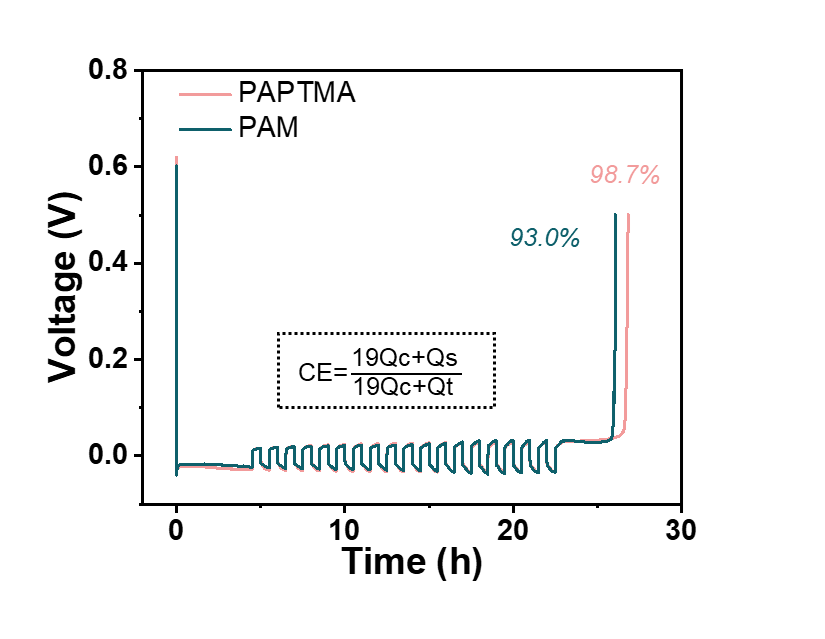


**Fig. S25** Determination of coulombic efficiency using Adams’s method


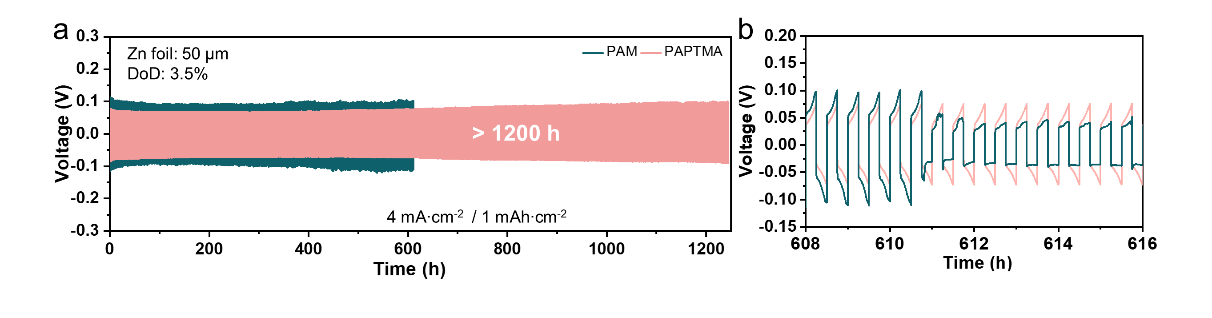


**Fig. S26** Long-term cycling performance of symmetric cells at 4 mA∙cm^-2^. PAM symmetric cell failed at 611 h


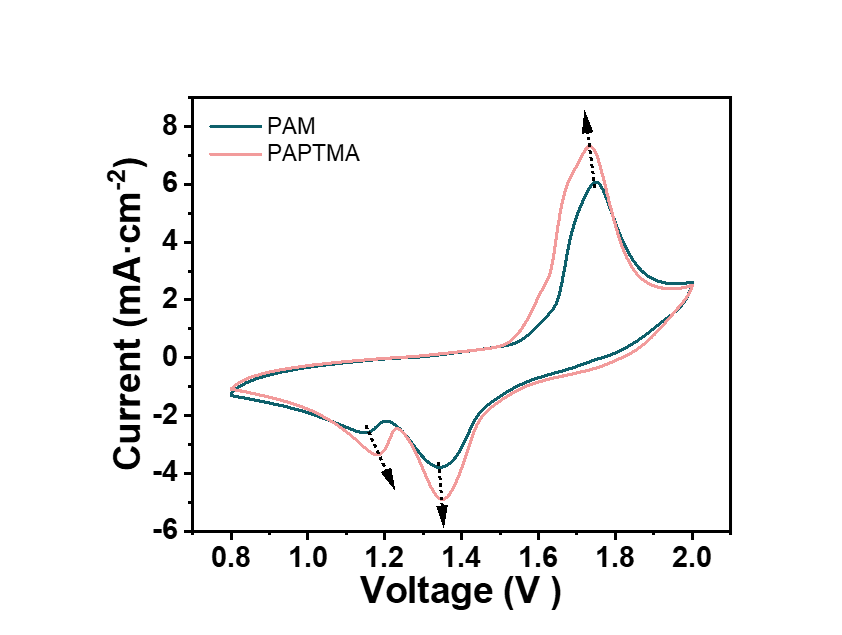


**Fig. S27** CV profiles of Zn|Gel|MnO_2_ cells


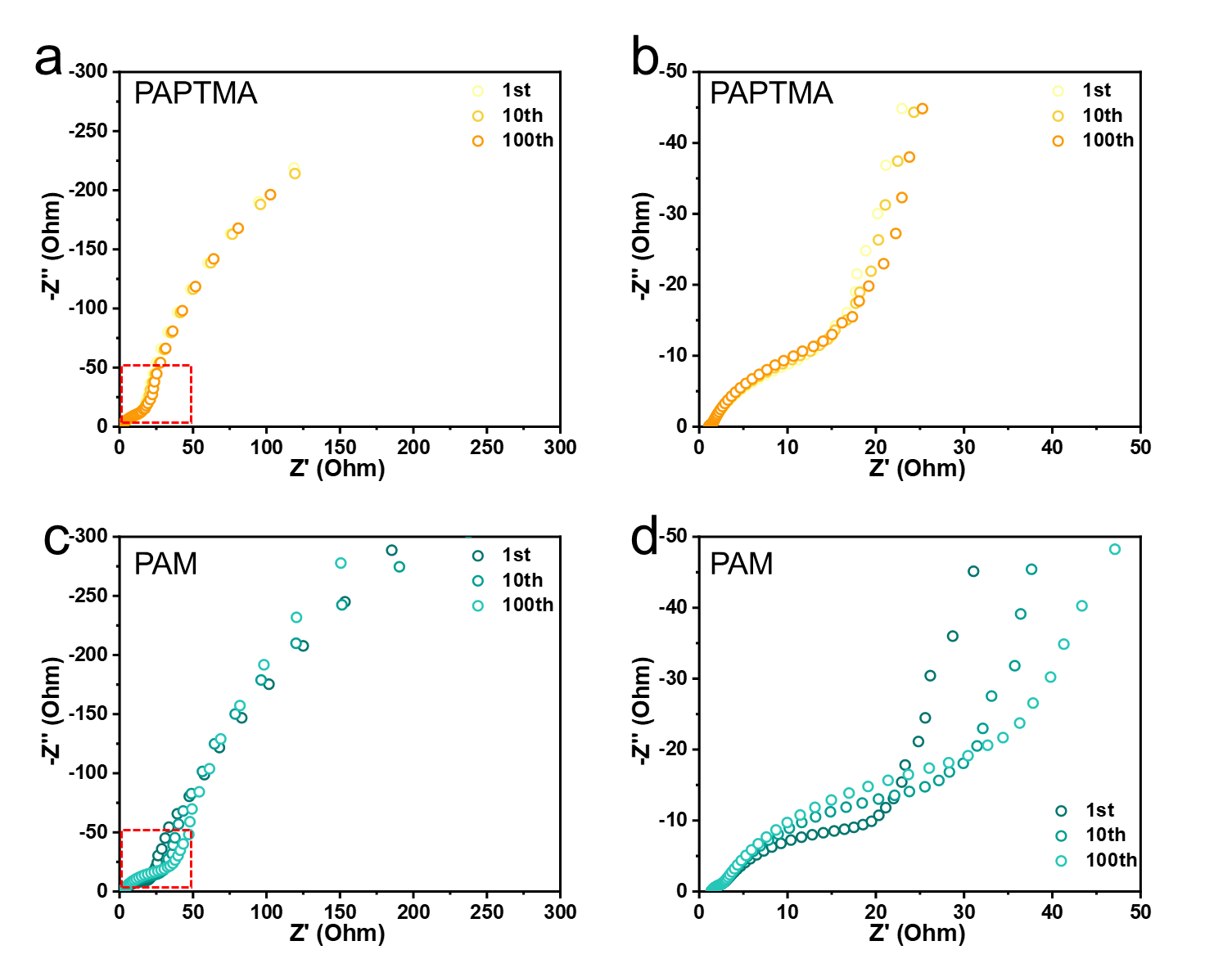


**Fig. S28** Ex situ EIS profiles of full cells using (**a, b**) PAPTMA and (**c, d**) PAM


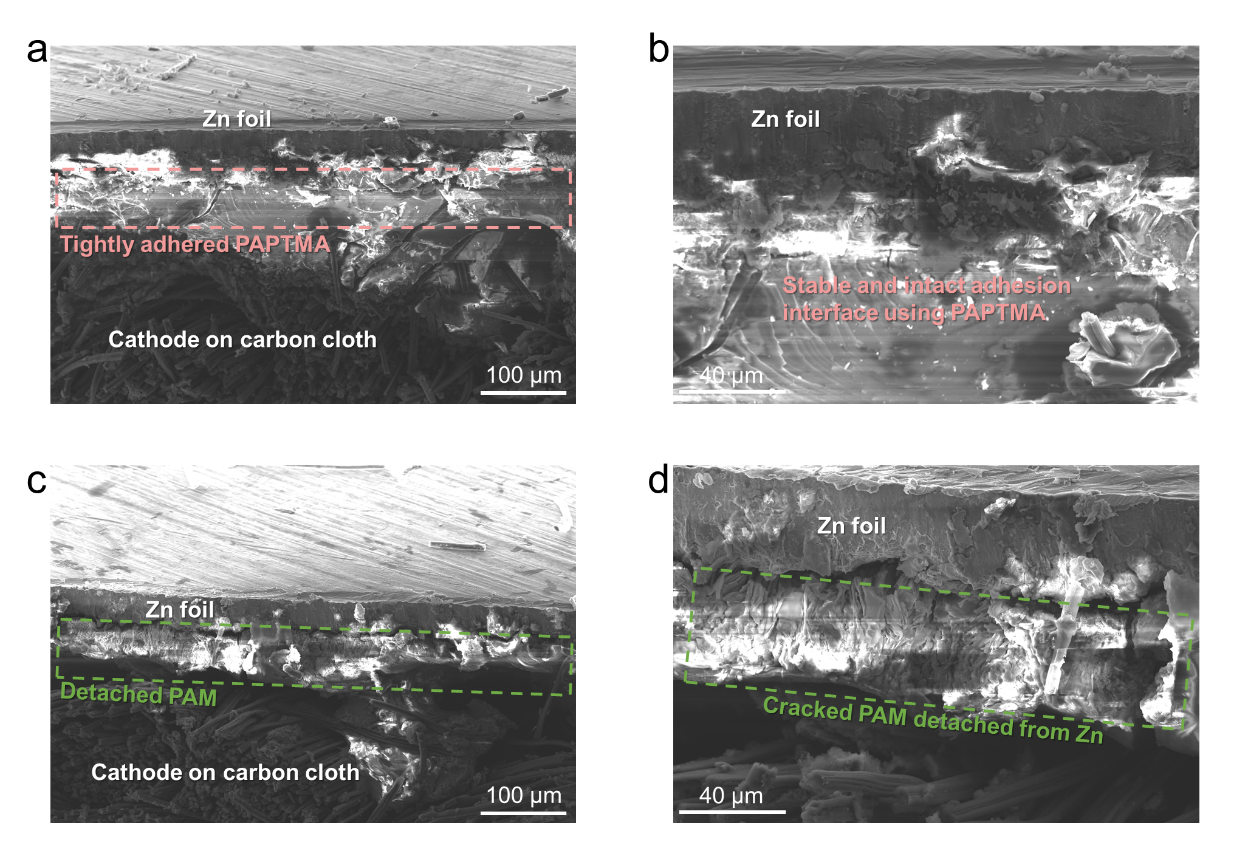


**Fig. S29** Cross sectional SEM images of (**a, b**) PAPTMA and (**c, d**) PAM after 100 cycles in full cells


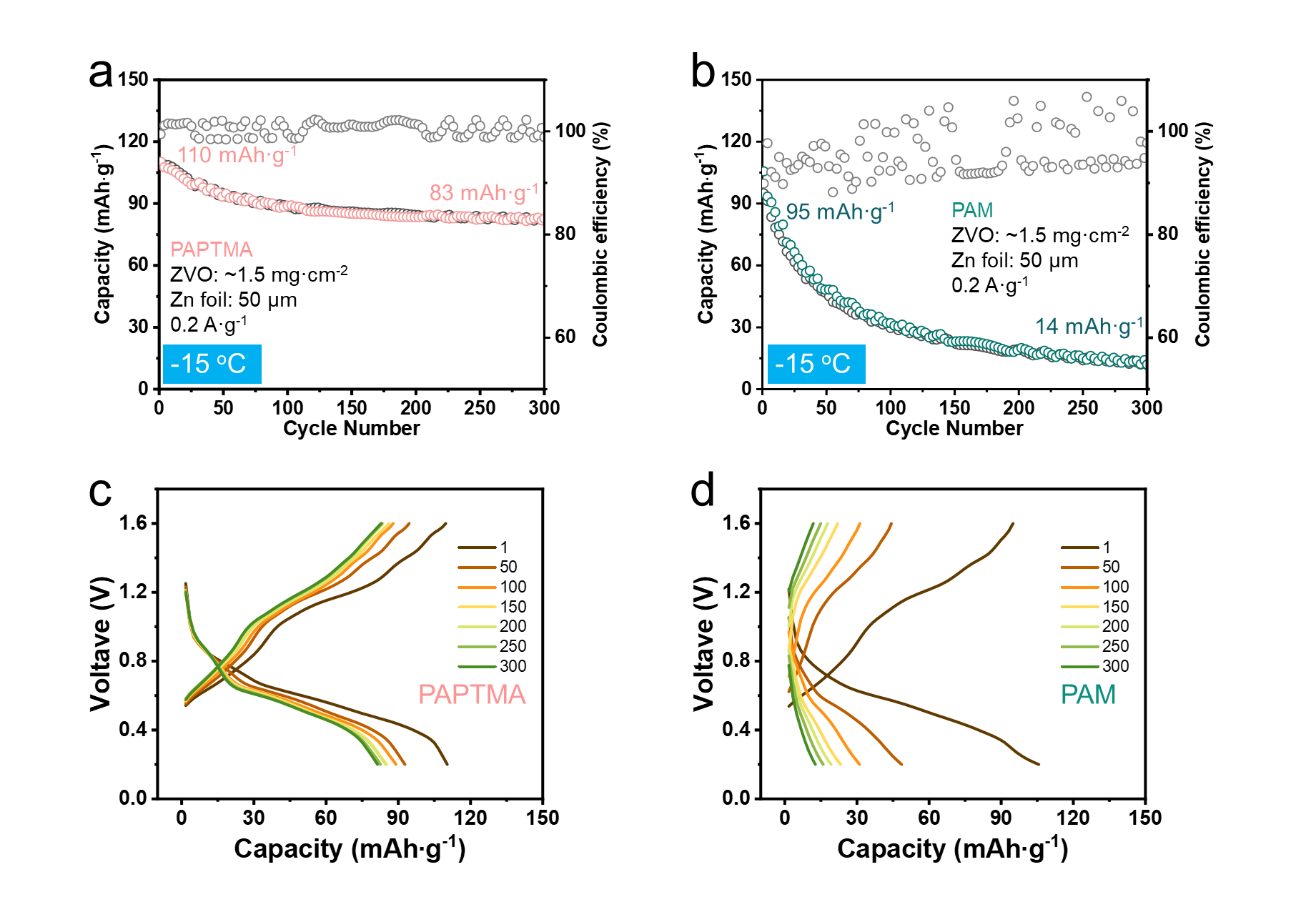


**Fig. S30** Full cell performance using PAPTMA (**a, c**) and PAM (**b, d**) at -15 ℃


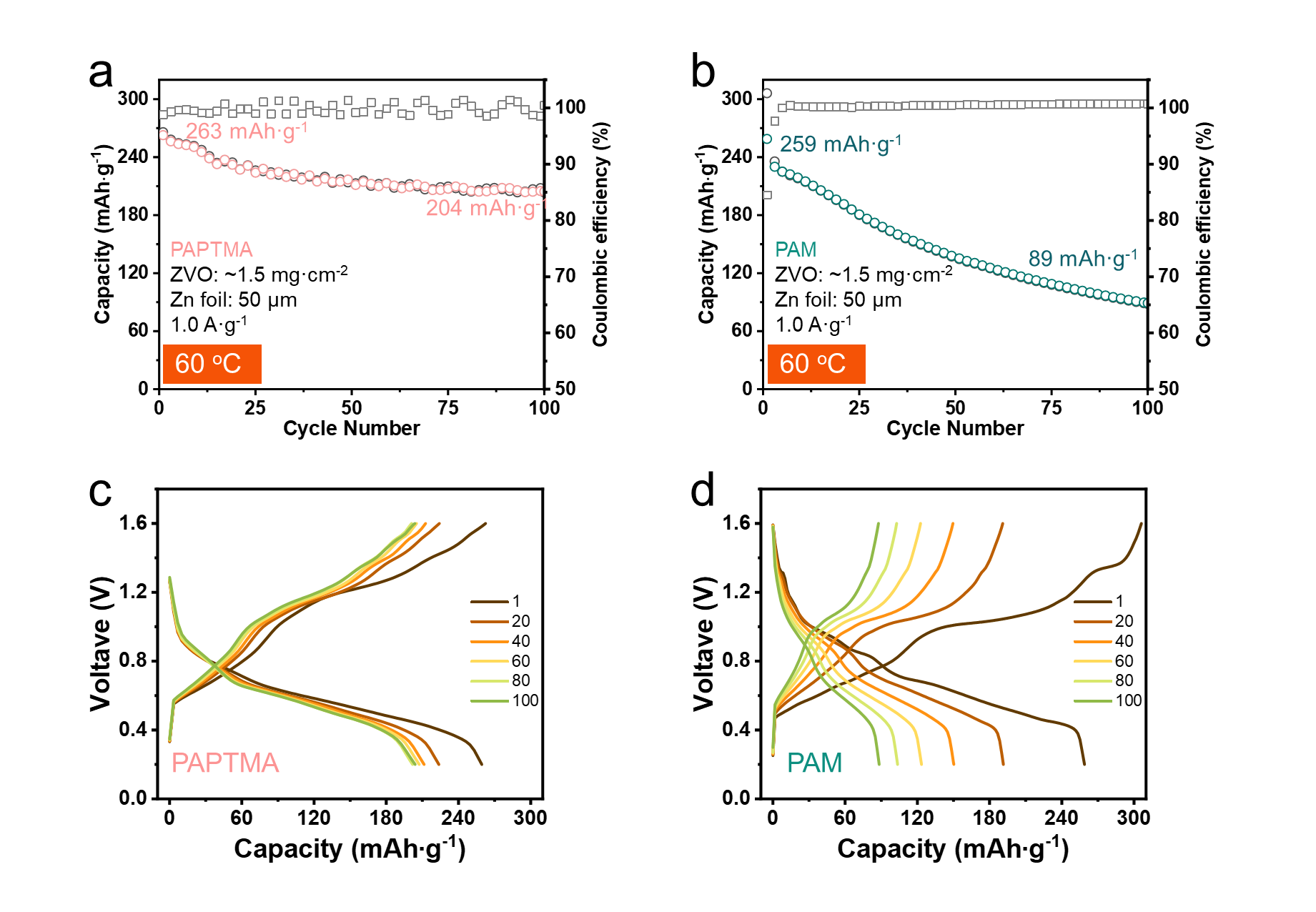


**Fig. S31** Full cell performance using PAPTMA (**a, c**) and PAM (**b, d**) at 60 ℃


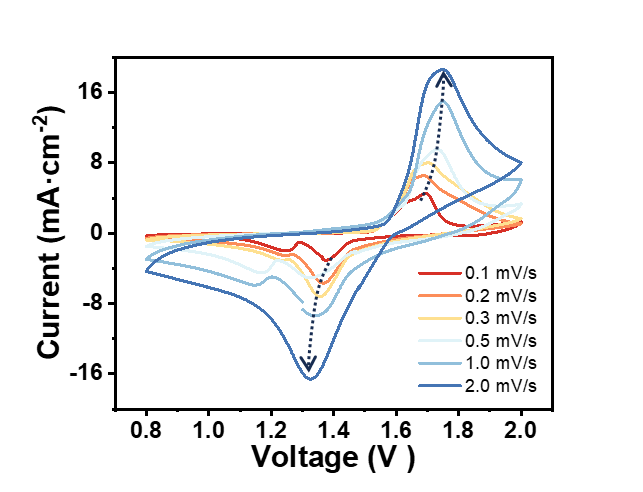


**Fig. S32** CV curves of pouch batteries based on PAPTMA hydrogel electrolytes with increasing scan rate


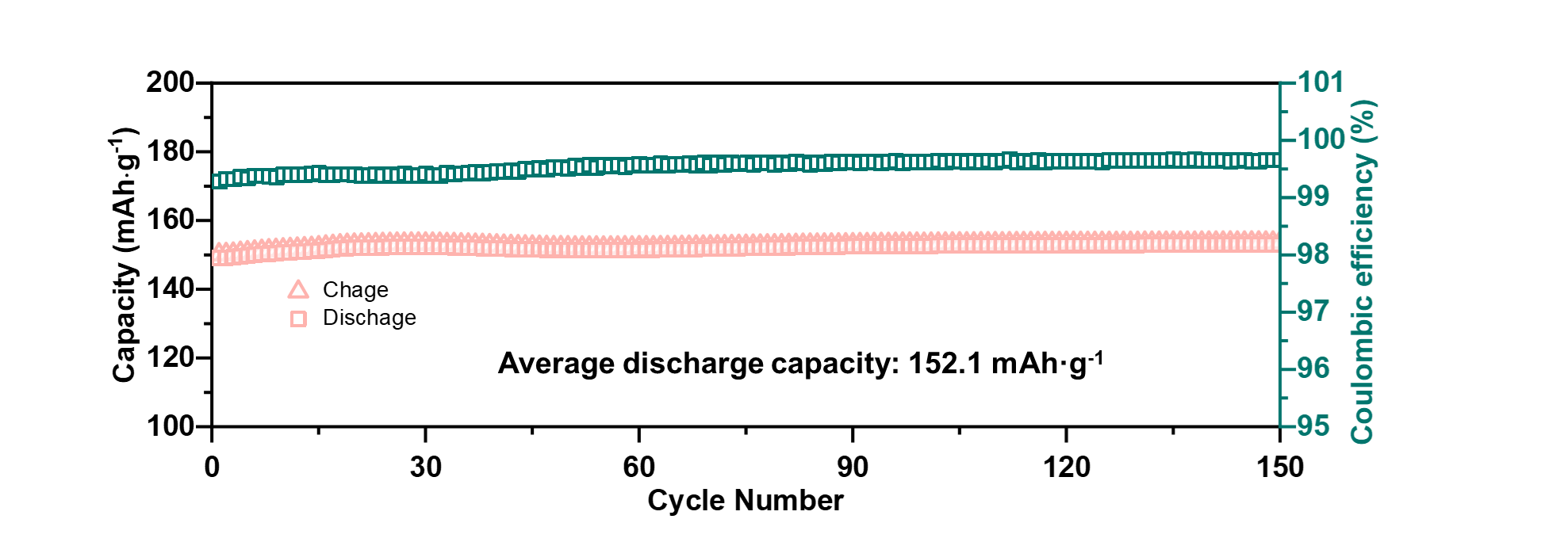


**Fig. S33** Cycling performance of pouch batteries under 0.5 A·g^-1^

**Table S1** The amount of each substance for the preparation of hydrogel electrolytes.

|  | PAPTMA  (3.5 M) | APTMA:AM =9:1 | APTMA:AM=7:3 | APTMA:AM=5:5 | PAM  (3.5 M) | PAPTMA  (2.0 M) | PAPTMA  (3.0 M) | PAPTMA  (4.0 M) | PAPTMA  (4.5 M) |
| --- | --- | --- | --- | --- | --- | --- | --- | --- | --- |
| **APTMA**  **(75% w/w) (g)** | **3.87** | **3.48** | **2.61** | **1.93** | **\** | **2.21** | **3.29** | **4.42** | **4.98** |
| **AM (g)** | **\** | **0.1** | **0.3** | **0.5** | **1** | **\** | **\** | **\** | **\** |
| **H_2_O (mL)** | **3.03** | **3.13** | **3.35** | **3.52** | **4.00** | **3.45** | **3.18** | **2.89** | **2.76** |
| **ZnSO_4_·7H_2_O (g)** | **2.30** | **2.30** | **2.30** | **2.30** | **2.30** | **2.30** | **2.30** | **2.30** | **2.30** |
| **MBA (mg)** | **20** | **20** | **20** | **20** | **20** | **11.4** | **17.1** | **22.9** | **25.7** |
| **APS**  **(5% w/v) (μL)** | **100** | **100** | **100** | **100** | **100** | **57** | **86** | **114** | **129** |
| **TEMED (μL)** | **10** | **10** | **10** | **10** | **10** | **5.7** | **8.6** | **11.4** | **12.9** |

Note: Monomer concentration is set according to the volume of water.

**Table S2** Calculation details of Zn^2+^ transference number

|  | **PAM** | **PAPTMA** |
| --- | --- | --- |
| ∆V (mV) | 10 | 10 |
| I_0_ (μA) | 13.0 | 12.7 |
| I_s_ (μA) | 4.7 | 9.0 |
| R_0_ (Ω) | 316 | 243 |
| R_s_ (Ω) | 843 | 418 |
| t$\text{Zn}^{\text{2+}}$ | 0.36 | **0.79** |

**Table S3** Comparison of hydrogel performance with reported works

| Material | Current density  (mA cm^-2^) | Areal  capacity (mAh cm^-2^) | Cycle life | Ionic conductivity (mS cm^−1^) | ***t***$\text{Zn}^{\text{2+}}$ | References |
| --- | --- | --- | --- | --- | --- | --- |
| P(AM-*co*-SBMA) | 2 | 2 | 400 | 32.9 | 0.84 | *Adv. Energy Mater.* **2022**, 12, 2202219 |
| Hyaluronic Acid | 1 | 1 | 5500 | 47.7 | 0.73 | *Nat Commun.* **2023**, 14, 6526 |
| Cellulose-CMC | 40 | 8 | 440 | 26 | 0.39 | *Adv. Funct. Mater.* **2023**, 33, 2302098 |
| TCOF-S-PAM | 1 | 0.5 | 1100 | 27.2 | 0.89 | *Angew. Chem. Int. Ed.* **2023**, 62, e202312020. |
| CD-PEO/PAM | 1 | 1 | 160 | 22.4 | 0.92 | *Adv. Mater.* **2023**, 35, 2301996 |
| PSX | 0.1 | 0.1 | 2000 | 18.9 | 0.80 | *Energy Storage Mater.* 2022, 51, 588. |
| CCH | 1 | 1 | 5000 | 9.7 | 0.81 | *Adv. Mater.* **2024**, 36, e2313610. |
| SFPAM-Zr | 0.5 | 0.5 | 2500 | 22.9 | 0.71 | *Energy Environ. Sci.*, **2023**,16, 4561-4571 |
| R-ZSO | 2 | 2 | 1500 | 30.3 | 0.59 | *Angew. Chem. Int. Ed.* **2024**, 63, e202318928 |
| Kevlar H | 1 | 1 | 3500 | 9.9 | 0.69 | *ACS Energy Lett.* **2023**, 8, 4, 1959 |
| Zinc Alginate | 2 | 0.25 | 2200 | 0.54 | 0.82 | *Adv. Funct. Mater.* **2023**, 33, 2300952 |
| CSAM | 1 | 1 | 700 | 25.2 | 0.76 | *Adv. Mater.* **2022**, 34, 2110140 |
| OR-PUU/PAM | 1 | 1 | 2000 | 26.9 | 0.78 | *Adv. Mater.* **2024***,* 36, 2311082 |
| C-PVA/PAN | 1 | 1 | 3400 | 17.4 | 0.64 | *Energy Environ. Sci.,* **2024***,* 10.1039/d4ee01993h |
| Electropolymerized hydrogel | 1 | 1 | 6060 | 10.6 | 0.77 | *Angew. Chem. Int. Ed.* **2024**, 21, e202400230 |
| PAPTMA | 1  4  8 | 1  1  1 | 6070  1240  500 | 28.7 | 0.79 | ***This work*** |

**Supplementary References**

1. T.D. Kühne, M. Iannuzzi, M. Del Ben, V.V. Rybkin, P. Seewald et al., CP2K: an electronic structure and molecular dynamics software package - quickstep: efficient and accurate electronic structure calculations. J. Chem. Phys. **152**(19), 194103 (2020). <https://doi.org/10.1063/5.0007045>
2. J. VandeVondele, J. Hutter, Gaussian basis sets for accurate calculations on molecular systems in gas and condensed phases. J. Chem. Phys. **127**(11), 114105 (2007). <https://doi.org/10.1063/1.2770708>
3. J.P. Perdew, K. Burke, M. Ernzerhof, Generalized gradient approximation made simple. Phys. Rev. Lett. **77**(18), 3865–3868 (1996). <https://doi.org/10.1103/physrevlett.77.3865>
4. S. Grimme, J. Antony, S. Ehrlich, H. Krieg, A consistent and accurate *ab initio* parametrization of density functional dispersion correction (DFT-D) for the 94 elements H-Pu. J. Chem. Phys. **132**(15), 154104 (2010). <https://doi.org/10.1063/1.3382344>
